# Radiomic Features of Multi-ROI and Multi-Phase MRI for the Prediction of Microvascular Invasion in Solitary Hepatocellular Carcinoma

**DOI:** 10.3389/fonc.2021.756216

**Published:** 2021-10-07

**Authors:** Yan Yang, WeiJie Fan, Tao Gu, Li Yu, HaiLing Chen, YangFan Lv, Huan Liu, GuangXian Wang, Dong Zhang

**Affiliations:** ^1^ Department of Radiology, Second Affiliated XinQiao Hospital of Army Medical University, ChongQing, China; ^2^ Department of Pathology, Second Affiliated XinQiao Hospital of Army Medical University, ChongQing, China; ^3^ GE Healthcare, Shanghai, China; ^4^ Department of Radiology, People’s Hospital of Banan District, ChongQing, China

**Keywords:** hepatocellular carcinoma, microvascular invasion, magnetic resonance imaging, radiomics analysis, nomogram

## Abstract

**Objectives:**

To develop and validate an MR radiomics-based nomogram to predict the presence of MVI in patients with solitary HCC and further evaluate the performance of predictors for MVI in subgroups (HCC ≤ 3 cm and > 3 cm).

**Materials and Methods:**

Between May 2015 and October 2020, 201 patients with solitary HCC were analysed. Radiomic features were extracted from precontrast T_1_WI, arterial phase, portal venous phase, delayed phase and hepatobiliary phase images in regions of the intratumoral, peritumoral and their combining areas. The mRMR and LASSO algorithms were used to select radiomic features related to MVI. Clinicoradiological factors were selected by using backward stepwise regression with AIC. A nomogram was developed by incorporating the clinicoradiological factors and radiomics signature. In addition, the radiomic features and clinicoradiological factors related to MVI were separately evaluated in the subgroups (HCC ≤ 3 cm and > 3 cm).

**Results:**

Histopathological examinations confirmed MVI in 111 of the 201 patients (55.22%). The radiomics signature showed a favourable discriminatory ability for MVI in the training set (AUC, 0.896) and validation set (AUC, 0.788). The nomogram incorporating peritumoral enhancement, tumour growth type and radiomics signature showed good discrimination in the training (AUC, 0.932) and validation sets (AUC, 0.917) and achieved well-fitted calibration curves. Subgroup analysis showed that tumour growth type was a predictor for MVI in the HCC ≤ 3 cm cohort and peritumoral enhancement in the HCC > 3 cm cohort; radiomic features related to MVI varied between the HCC ≤ 3 cm and HCC > 3 cm cohort. The performance of the radiomics signature improved noticeably in both the HCC ≤ 3 cm (AUC, 0.953) and HCC > 3 cm cohorts (AUC, 0.993) compared to the original training set.

**Conclusions:**

The preoperative nomogram integrating clinicoradiological risk factors and the MR radiomics signature showed favourable predictive efficiency for predicting MVI in patients with solitary HCC. The clinicoradiological factors and radiomic features related to MVI varied between subgroups (HCC ≤ 3 cm and > 3 cm). The performance of radiomics signature for MVI prediction was improved in both the subgroups.

## Introduction

Hepatocellular carcinoma (HCC) represents a major public health problem worldwide. Currently, liver transplantation, surgical resection and radiofrequency ablation are established treatments for early-to-intermediate stage HCC, among which surgical resection remains the mainstay of curative treatment (1, 2). Nevertheless, HCC is refractory to therapeutic interventions, largely because HCC has a propensity to invade blood vessels and thus spread intrahepatically and/or extrahepatically *via* tumour emboli (1–3). The presence of microvascular invasion is a critical determinant of early recurrence and poor prognosis based on the results of multiple retrospective studies (4–6).

Microvascular invasion (MVI) is defined as the presence of tumour cells within a vascular space lined by endothelium, and can only be reliably determined on histopathological examinations of resected surgical specimens (3). Multiple research teams have revealed that MVI occurs in 15% to 74.4% of resected specimens and is independently related to early recurrence and poor overall survival (7–10). It has been previously suggested that a wider resection margin should be performed in the presence of MVI, even for a small lesion; in addition, additional adjuvant therapies after resection might be preferable (11, 12). Therefore, the knowledge of MVI status at the time of an HCC diagnosis would be of great help for physicians to make more informed management decisions and thus to improve prognostication. Several radiological features have previously been suggested as predictors for MVI, including a larger tumour size (13, 14), a nonsmooth tumour margin (5, 13, 15, 16), an absent or incomplete radiological capsule (14, 17, 18), peritumoral enhancement on contrast-enhanced CT or MRI (13, 15–18), and peritumoral hypointensity on the hepatobiliary phase (HBP) images (16, 19).

Radiomics is a brand-new imaging analysis technique that can transform medical images into innumerable quantitative features (20). Recent studies have confirmed that imaging features extracted from Gd-EOB-DTPA-enhanced MRI have a high value in the prediction of MVI in patients with HCC (15, 21). Moreover, the combination of the radiomics signature derived from Gd-EOB-DTPA enhanced MRI images with clinicoradiological risk factors could improve the predictive efficacy for MVI (15). Pathologically, MVI is frequently found in the small vessels (including the portal vein, the hepatic vein, and occasionally the hepatic artery, bile duct, and lymphatic vessels) in the adjacent liver tissues of tumours (22). Taking this into account, we may assume that the radiomics signature abstracted from the peritumoral region may reveal a more direct association with MVI. In the study by Feng et al., the MVI prediction performance of the radiomics signature extracted from the intratumoral and peritumoral areas of the HBP images was superior to that extracted from only the intratumoral area (21). However, they did not estimate the prediction performance of radiomics features extracted from other phases, such as the arterial and portal venous phases, on which certain radiological features (e.g., peritumoral enhancement and the absence of radiological capsules) have been shown to be associated with MVI.

Because the incidence of MVI increases with tumour size, a larger tumour size has historically been considered a risk factor for the presence of MVI in patients with HCC (23–26). However, the usefulness of tumour size alone and the appropriate cut-off value of tumour size for predicting MVI have been the subjects of ongoing debates, which may be due to the selection bias of study designs or surgical candidates. In subgroup analyses (tumours ≤ 2 cm, 2–5 cm, and >5 cm), Matteo et al. found that imaging features, such as nonsmooth tumour margins, peritumoral enhancement, and the two-trait predictor of venous invasion (TTPVI), were more useful for smaller tumours, whereas for larger tumour, size had a greater weight on MVI prediction (13). Many studies have suggested that HCC may reach an important turning point in critical transformation when tumours grow to a size of 3 cm, leading to more invasive behaviour (27, 28). The study by Sudeep et al. pointed out that radio-genomic venous invasion (RVI) had less discriminating power for tumours larger than 3 cm than for tumours smaller than 3 cm (29). These studies suggested that there might be differences in the predictors for MVI between HCCs ≤ 3 cm and > 3 cm. However, to the best of our knowledge, there is no research that has explored and compared the predictive features, especially the radiomics features, for MVI in HCCs ≤ 3 cm and >3 cm.

In the present study, we sought to develop and validate a radiomics nomogram that would combine the radiomics signature and clinicoradiological risk factors for the preoperative prediction of MVI in patients with solitary HCC. Furthermore, we evaluated the predictive performance of the radiomics signature and clinicoradiological factors for MVI in HCC subgroups divided by using a tumour size of 3 cm as the cut-off value.

## Materials and Methods

### Patients’ Data

Our Institutional Ethics Review Board approved the current retrospective analysis of anonymous data and waived patient informed consent. Between May 2015 and October 2020, patients who were pathologically diagnosed with primary HCCs and underwent Gd-EOB-DTPA-enhanced MRI examinations were consecutively included in this study. The inclusion criteria were as follows: 1) patients with a single primary HCC; 2) patients who underwent curative hepatectomy; 3) patients who received Gd-EOB-DTPA enhanced MRI scan within 1 month before surgery; and 4) patients with histologically confirmed HCCs with full descriptions in the histopathologic reports. The exclusion criteria were as follows: 1) patients with gross vascular invasions or extrahepatic metastasis; 2) patients with a history of any anticancer therapy before surgery; and 3) patients with inadequate image quality for analysis. Finally, a total of 201 HCC patients (165 males and 36 females; mean age, 52.40 ± 10.38 years) were enrolled in this study. According to the date of the MRI scan, the cohort was divided into a training set (n = 148; 125 males and 23 females; from May 2015 to December 2017) and a validation set (n = 53; 40 males and 13 females; from January 2018 to October 2020) at a ratio of 7: 3. In addition, the primary cohort was reassigned into two subgroups: the HCCs ≤ 3 cm cohort (n =94; 76 males and 18 females) and the HCCs > 3 cm cohort (n =107; 89 males and 18 females).

Demographic and clinical laboratory data were collected from medical records, including age, sex, serum alpha-fetoprotein (AFP) levels, serum alanine aminotransferase (ALT) levels, aspartate aminotransferase (AST) levels, serum albumin (ALB) levels, total bilirubin (TBIL) levels, γ-glutamyl transpeptidase (GGT) levels, and prothrombin time (PT) levels. The pathological characteristics of the specimens from hepatectomy, particularly for MVI status, were assessed in consensus by 2 dedicated pathologists. MVI was defined as the presence of cancer cell nest in the portal vein, hepatic vein, or a large capsular vessel of the surrounding hepatic tissue lined with endothelium that was visible only on microscopy (22).

### MR Examination

All MRI scans were performed with a 1.5T or 3.0T MR scanner (Signa HDxt, GE Healthcare) equipped with a quadrature body coil [training set: 1.5T (n=130), 3.0T (n=19); validation set: 1.5T (n=25), 3.0T (n=28)]. Regions of interest (ROI) were drawn on axial LAVA (liver acquisition with volume acceleration) MR images, including precontrast T1-weighted images (T_1_WI), and arterial phase (AP, 20–35 s), portal venous phase (VP, 60–70 s), delayed phase (DP, 3 min) and hepatobiliary biliary phase (HBP, 20 min) images, after the injection of 0.025 mmol/kg of Gd-EOB-DTPA (Primovist, Bayer Schering Pharma, Berlin, Germany) into the cubital vein, followed by a 20 mL saline flush. These images were obtained by using the following parameters (1.5T/3.0T): TR = 3.8/2.6 ms, TE = 1.8/1.2 ms, flip angle =15/11°, thickness = 4.8/5.0 mm, FOV = 40/38 cm, and bandwidth =62.50/125 kHz. Other images, such as T2-weighted images (T_2_WI) and diffusion-weighted images (DWI), were also obtained, but they were not used for radiomic analyses, for the thickness of these images was about 6 to 10 mm, which might affect the quantification of radiomic feature (30, 31).

MR images were reviewed independently by two radiologists, Y. Yang (reader A) and G.X. Wang (reader B), with 5 and 10 years of experience in abdominal MR imaging interpretation, respectively. They were blinded to MVI status and other clinical information. The two radiologists independently assessed the following morphological features for each tumour: tumour size, tumour growth type, enhancement pattern, radiologic capsule, peritumoral enhancement, tumour signal intensity on the HBP image, peritumoral hypointensity on the HBP image, intratumoral fat, intratumoral necrosis, intratumoral haemorrhage, and intratumoral vasculature at the arterial phase (13, 16, 17, 19, 32, 33). Any disagreement in imaging feature assessment between the radiologists was settled by a joint review until a final consensus was reached.

### Tumour Segmentation and Radiomic Features Extraction

**Figure 1:**
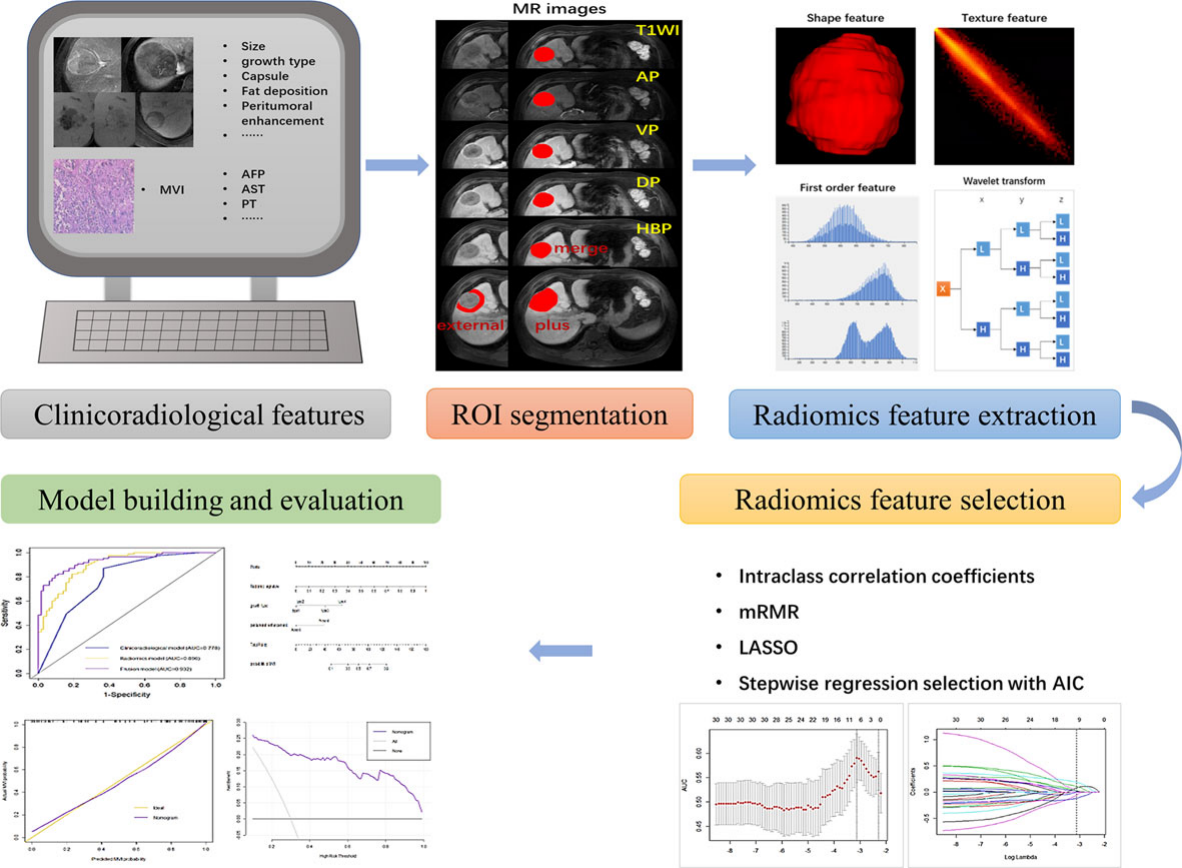
A flowchart showing the radiomics analysis for MVI prediction. The clinicoradiological characteristics (especially the status of MVI) were identified first. ROI segmentation was performed on axial LAVA MR images, and then radiomic features were extracted, including shape features, first-order features, textural features, and wavelet transformed features. Next, features with high stability (ICC > 0.8) were included and further selected *via* mRMR, LASSO and stepwise regression analysis with AIC. The MVI prediction model was constructed by incorporating the radiomics signature and clinicoradiological risk factors. A nomogram was adopted to present the model and evaluated with calibration curve and decision curve analysis.

The workflow is shown in **Figure 1**. First, all MR images were resampled to a voxel size of 1×1×1 mm^3^ by linear interpolation to standardize the voxel spacing. Regions of the entire intratumoral area (ROI-merge) were drawn semiautomatically with reference to the boundary of the tumour on the HBP image. To capture features from the peritumoral area of 1 cm (ROI-external), where there is more potential for microvascular invasion, a 1-cm-wide area was obtained with a dilation algorithm. The combined intratumoral and peritumoral area (ROI-plus) was generated synchronously. Of note, the nonhepatic regions of the ROI were subtracted semiautomatically or manually slice-by-slice, as appropriate. In addition, the voxel intensity values were discretized by using a fixed bin width of 5. A wavelet filter, which decomposed the original image into 8 decompositions, was implemented to extract high-dimensional features from different frequency scales. A total of 851 radiomic features, including first-order features, shape features, texture features, and wavelet-transformed features, were extracted from each three-dimensional ROI. Tumour segmentation and feature extraction were performed by reader A and reviewed by reader B. These steps were implemented by using AK software (Artificial-Intelligence Kits; version 3.3.0; GE Healthcare).

### Radiomic Features Selection and Signature Construction

We randomly selected 20 patients and repeated the same procedure one month later. The intraclass correlation coefficient (ICC) was calculated to determine the stability of the features. Features with ICCs lower than 0.80 were excluded, and the remaining features were used for subsequent evaluation. All the patients were divided into the training set and validation set according to the date of the MRI scan at a ratio of 7:3. In addition, all the patients were reassigned into HCC ≤ 3 cm and HCC > 3 cm cohorts. The abnormal values were replaced by the media and all the features were standardized before selection. Then, the minimum redundancy maximum relevance (mRMR), least absolute shrinkage and selection operator (LASSO) algorithm and stepwise logistic regression analysis with Akaike information criterion (AIC) were used to select MVI-related features. Receiver operating characteristic (ROC) curves were drawn to display their performance for MVI prediction. The area under the curve (AUC) and corresponding 95% confidence interval (CI) were obtained from ROC curves, as well as the sensitivity, specificity and accuracy. Multiple comparisons of ROC curves in the training set were performed by the Delong test with Bonferroni-adjusted *p* values, and independently validated in the validation set.

### Model Construction and Evaluation

Univariate and multivariate regression analyses with odds ratios (ORs) were performed to determine the MVI risk factors. The clinicoradiological model was formulated based on the results of multivariate regression. The radiomics signature and significant clinicoradiological risk factors were used to construct the combined model using multivariable logistic regression analysis in the training set. In view of the multivariable logistic regression, the collinearity diagnosis was performed using the variance inflation factor (VIF). The performances of the clinicoradiological model, radiomics model and the combined model in predicting MVI were tested using ROC analysis; the AUC with the corresponding 95% CI, sensitivity, specificity, and accuracy were calculated. We also established a nomogram for the combined prediction model to provide a more direct way for clinicians to assess the possibility of MVI. A calibration curve, a graphic representation of the relationship between the actual MVI and the predicted MVI probabilities, was plotted to assess the calibration of the nomogram. In addition, decision curve analysis (DCA) was also conducted to estimate the clinical utility of the nomogram by quantifying the net benefits for a range of high risk thresholds in the combined training and validation set (34).

### Statistical Analysis

The statistical software R (version 4.0.2) and Python (version 3.5.6) were used to perform the statistical analysis. Categorical variables are presented as whole numbers and proportions, and continuous variables are presented as medians with interquartile ranges. Clinicoradiological variables associated with MVI were assessed based on clinical importance and predictors identified in previously published articles (13–15, 17, 21, 32). The significant differences of the variables were analysed in the training and validation sets (MVI-presence group and MVI-absence group), as well as in the subgroups (HCC ≤ 3 cm and HCC > 3 cm), by using the chi-square test or the Fisher exact test for the categorical variables and the Mann-Whitney U test for the continuous variables. The associations of relevant clinicoradiological variables with MVI were assessed using binary logistic regression models. Backward stepwise selection with the Akaike information criterion (AIC) was used to identify variables for the multivariable logistic regression models. The packages in R that were used in this study were as follows: the “glmnet” package to perform the LASSO logistic regression model analysis, the “car” package to calculate the VIFs, the “epiDisplay” package to plot the ROC curves, the “rms” package to construct the nomogram and plot the calibration curves, and the “rmda” to perform DCA. A two-sided *p* < 0.05 was regarded as statistically significant.

## Results

### Clinicoradiological Characteristics and MVI Prediction Factors

Among the 201 patients with solitary HCC, MVI was diagnosed in the resected tissue of 111 patients (55.22%). The high prevalence of MVI observed in the present analysis might be the consequence of a more careful histological examination of surgical specimens. Although this prevalence seems to be high, it was reported to be highly variable in different series (10, 11). The clinicoradiological characteristics of patients in the training and validation sets are summarized in **Table 1**. There was no significant difference between the two sets regarding MVI status (*p* = 0.293). The tumour size of HCC with MVI was significantly larger than that of HCC without MVI in both sets (*p* < 0.05). HCC patients with and without MVI demonstrated significantly different imaging features. Tumour growth type, peritumoral enhancement and peritumoral hypointensity on HBP images were significantly associated with MVI in patients with solitary HCC (*p* < 0.001). Tumour size, serum AFP levels, tumour capsules, intratumoral vasculature and necrosis were also related to the presence of MVI (*p* < 0.05). Backward stepwise selection using the AIC in multivariate logistic regression analysis modelling confirmed that peritumoral enhancement and tumour growth type had the strongest associations with the presence of MVI in the training set (**Table 2**), and these two factors were used for the clinicoradiological model construction.

**Table 1:** Patient characteristics in the training and validation sets.

Variables	Training set			Validation set		
	MVI absence n = 63	MVI presence n = 85	*P value*	MVI absence n = 27	MVI presence n = 26	*P value*
Age (year)	50.00 (45.00, 57.00)	52.00 (47.00, 61.00)	0.191	52.00 (46.00, 54.00)	51.00 (46.00, 61.50)	0.650
Sex			0.923			0.810
Female	10 (15.87%)	13 (15.29%)		7 (25.93%)	6 (23.08%)	
Male	53 (84.13%)	72 (84.71%)		20 (74.07%)	20 (76.92%)	
AFP level (ng/ml)			0.021			0.785
<20	31 (49.21%)	25 (29.41%)		14 (51.85%)	11 (42.31%)	
20–400	17 (26.98%)	23 (27.06%)		7 (25.93%)	8 (30.77%)	
>400	15 (23.81%)	37 (43.53%)		6 (22.22%)	7 (26.92%)	
ALT level (U/L)			0.264			0.922
<40	32 (50.79%)	51 (60.00%)		18 (66.67%)	17 (65.38%)	
>40	31 (49.21%)	34 (40.00%)		9 (33.33%)	9 (34.62%)	
AST level (U/L)			0.588			0.132
<35	35 (55.56%)	51 (60.00%)		18 (66.67%)	12 (46.15%)	
>35	28 (44.44%)	34 (40.00%)		9 (33.33%)	14 (53.85%)	
ALB level (g/L)			0.234			1.000
>40	53 (84.13%)	77 (90.59%)		22 (81.48%)	21 (80.77%)	
<40	10 (15.87%)	8 (9.41%)		5 (18.52%)	5 (19.23%)	
T-BIL level (μmol/l)			0.103			0.340
<20	43 (68.25%)	68 (80.00%)		18 (66.67%)	14 (53.85%)	
>20	20 (31.75%)	17 (20.00%)		9 (33.33%)	12 (46.15%)	
ALP level (U/L)			0.864			0.351
<135	55 (87.30%)	75 (88.24%)		26 (96.30%)	23 (88.46%)	
>135	8 (12.70%)	10 (11.76%)		1 (3.70%)	3 (11.54%)	
GGT level (U/L)			0.621			0.498
<45	27 (42.86%)	33 (38.82%)		16 (59.26%)	13 (50.00%)	
>45	36 (57.14%)	52 (61.18%)		11 (40.74%)	13 (50.00%)	
PT (s)			1.000			1.000
<14	62 (98.41%)	83 (97.65%)		26 (96.30%)	25 (96.15%)	
>14	1 (1.59%)	2 (2.35%)		1 (3.70%)	1 (3.85%)	
**MR features**						
Tumour size (cm)	29.00 (20.00,52.00)	45.00 (28.50,62.50)	0.003	23.00 (19.00,28.00)	29.00 (22.25.55.75)	0.017
Tumour growth type			<0.001			<0.001
Smooth regular nodule growth	7 (11.11%)	1 (1.11%)		3 (11.11%)	2 (7.69%)	
Focal extranodular growth	15 (23.81%)	8 (9.41%)		13 (48.15%)	1 (3.85%)	
Multinodular confluent growth	29 (46.03%)	26 (30.59%)		8 (29.63%)	10 (38.46%)	
Infiltrative growth	12 (19.05%)	50 (58.82%)		3 (11.11%)	13 (50.00%)	
Tumour capsule			0.011			0.625
Absent	19 (30.16%)	32 (37.65%)		11 (40.74%)	13 (50.00%)	
Incomplete	25 (39.68%)	44 (51.76%)		10 (37.04%)	10 (38.46%)	
Complete	19 (30.16%)	9 (10.59%)		6 (22.22%)	3 (11.54%)	
Enhancement pattern			0.803			1.000
Untypical	8 (12.7%)	12 (14.12%)		3 (11.11%)	3 (11.54%)	
Typical	55 (87.3%)	73 (85.88%)		24 (88.89%)	23 (88.46%)	
Peritumoral enhancement			<0.001			0.019
Absent	41 (65.08%)	18 (21.18%)		20 (74.07%)	11 (42.31%)	
Present	22 (34.92%)	67 (78.82%)		7 (25.93%)	15 (57.69%)	
HBP signal intensity			0.090			0.250
Other	12 (19.05%)	8 (9.41%)		2 (7.41%)	5 (19.23%)	
Hypointensity	51 (80.95%)	77 (90.59%)		25 (92.59%)	21 (80.77%)	
Peritumoral hypointensity on HBP			<0.001			0.300
Absent	47 (74.60%)	32 (37.65%)		22 (81.48%)	18 (69.23%)	
Present	16 (25.40%)	53 (62.35%)		5 (18.52%)	8 (30.77%)	
Intratumoral vasculature			0.013			0.003
Absent	44 (69.84%)	42 (49.41%)		22 (81.48%)	11 (42.31%)	
Present	19 (30.16%)	43 (50.59%)		5 (18.52%)	15 (57.69%)	
Intratumoral fat			0.225			0.691
Absent	45 (71.43%)	68 (80.00%)		21 (77.78%)	19 (73.08%)	
Present	18 (28.57%)	17 (20.00%)		6 (22.22%)	7 (26.92%)	
Intratumoral necrosis			0.004			0.026
Absent	44 (69.84%)	39 (45.88%)		23 (85.19%)	15 (57.69%)	
Present	19 (30.16%)	46 (54.12%)		4 (14.81%)	11 (42.31%)	
Intratumoral haemorrhage			0.082			0.351
Absent	51 (80.95%)	58 (68.24%)		26 (96.30%)	23 (88.46%)	
Present	12 (19.05%)	27 (31.76%)		1 (3.70%)	3 (11.54%)	

MVI, microvascular invasion; AFP, α-fetoprotein; ALT, alanine aminotransferase; AST, aspartate aminotransaminase; ALB, albumin; T-BIL, total bilirubin; ALP, alkaline phosphatase; GGT, γ-glutamyltransferase; PT, prothrombin time.

**Table 2:** Logistic regression analysis showing the association of variables with MVI presence in the training set.

Variables	Univariate analysis		Multivariate analysis	
	OR (95% CI)	*P value*	OR (95% CI)	*P value*
Age (year)	1.03 (0.99–1.06)	0.116		
Sex, Female *vs*. Male	0.96 (0.39–2.40)	0.923		
AFP level (ng/ml)				
<20	[Reference]			
20–400	1.68 (0.74–3.85)	0.216		
>400	3.06 (1.40–6.93)	0.006		
ALT level (IU/L),<40 *vs*.>40	0.69 (0.36–1.33)	0.256		
AST level (IU/L),<35 *vs*.>35	0.83 (0.43–1.61)	0.588		
ALB level (g/L),>40 *vs*.<40	0.55 (0.20–1.49)	0.239		
T-BIL level (μmol/l),<20 *vs*.>20	0.54 (0.25–1.14)	0.105		
ALP level (U/L),<135 *vs*.>135	0.92 (0.34–2.54)	0.864		
GGT level (U/L),<45 *vs*.>45	1.18 (0.61–2.30)	0.621		
PT (s),<14 *vs*.>14	1.49 (0.14–32.57)	0.745		
**MR features**				
Tumour size (cm)	1.02 (1.01–1.04)	0.005		
Tumour growth type				
Smooth regular nodule growth	[Reference]		[Reference]	
Focal extranodular growth	3.73 (0.52–76.26)	0.254	4.15 (0.53–89.11)	0.235
Multinodular confluent growth	6.28 (1.02–121.45)	0.096	4.82 (0.71–97.25)	0.168
Infiltrative growth	29.17 (4.60–573.18)	0.003	15.73 (2.21–322.58)	0.017
Capsule				
Complete	[Reference]			
Incomplete	3.72 (1.50–9.81)	0.006		
Absent	3.56 (1.37–9.78)	0.011		
Enhancement pattern,	0.88 (0.33–2.29)	0.803		
Untypical *vs*. Typical				
Peritumoral enhancement,	6.94 (3.39–14.78)	<0.001	4.38 (1.98–9.95)	0.003
Absent *vs*. Present				
HBP signal intensity,	2.26 (0.88–6.15)	0.096		
Hypointensity *vs*. Other				
Peritumoral hypointensity on HBP,	4.87 (2.42–10.20)	<0.001		
Absent *vs*. Present				
Intratumoral vasculature,	2.37 (1.21–4.78)	0.014		
Absent *vs*. Present				
Intratumoral fat,	0.63 (0.29–1.34)	0.227		
Absent *vs*. Present				
Intratumoral necrosis,	2.73 (1.39–5.51)	0.004		
Absent *vs*. Present				
Intratumoral haemorrhage,	1.98 (0.93–4.43)	0.085		
Absent *vs*. Present				

MVI, microvascular invasion; OR, odds ratio; AFP, α-fetoprotein; ALT, alanine aminotransferase; AST, aspartate aminotransaminase; ALB, albumin; T-BIL, total bilirubin; ALP, alkaline phosphatase; GGT, γ-glutamyltransferase; PT, prothrombin time.

### Radiomic Features Selection and Performance for MVI Prediction

**Figure 2:**
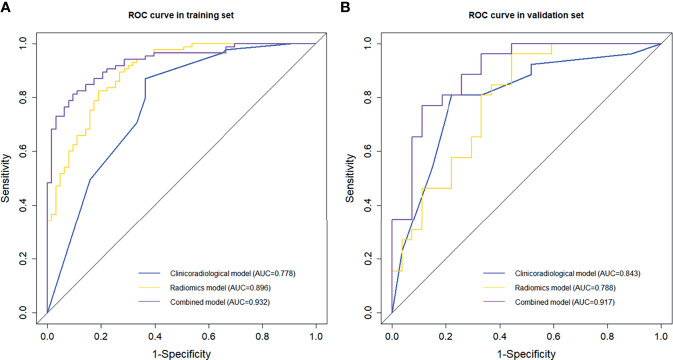
Comparison of receiver operating characteristic (ROC) curves in predicting MVI presence. ROC curves of the clinicoradiological model, radiomics model and the combined model in the **(A)** training and **(B)** validation sets.

The radiomic features selection using LASSO binary logistic regression analysis in the training set is shown in **Supplemental Figure 1**. The finally selected features of the ROI in a single phase are displayed in **Supplemental Table 1**. We established radiomic models based on the ROI of a single phase separately, and their corresponding AUCs and 95% confidence intervals (CIs), sensitivity and specificity are shown in **Supplemental Table 2** and **Figure 2**. For the vast majority of the ROI and phases, the performance of the ROI in AP and VP were superior to other phases, and the ROI-merge showed a superior performance compared with other ROI of their phases. Of note, features extracted from the ROI-external showed a stable performance in all phases. To integrate the relevant features in both intratumoral and peritumoral areas to improve the characterization of MVI in HCC patients, the optimal ROI in each phase was selected to build the radiomics model. A formula was generated using a linear combination of selected features that were weighted by their respective logistic regression coefficients, and then used to calculate the radiomics score (a risk score reflecting the probability of MVI) for each selected ROI. The final radiomics score was calculated for each patient *via* a linear combination of radiomics scores of selected ROIs weighted by their respective coefficients.

Herein, the ROI with optimal performance in each phase (i.e., ROI-merge in AP, DP, VP, and ROI-external in HBP, T_1_WI) were selected and incorporated into the formula to calculate the radiomics score. The radiomics score calculation formula is presented in **Supplemental Formula 1**. Patients in the MVI-presence group generally displayed a higher radiomics score [median (interquartile range)] than the patients in the MVI-absence group in the training set [1.750 (0.665 – 3.109) *vs*. -1.430 (-3.275 – 0.094), *p <*0.001] and in the validation set [0.447 (-1.991 – 1.372) *vs*.-0.837 (-2.098 – 0.340), *p* < 0.001].

### Model Comparison and Nomogram Construction and Evaluation

A combined model that incorporated clinicoradiological predictors (peritumoral enhancement and tumour growth type) and the radiomics signature was constructed. Collinearity tests showed that the VIFs ranged from 1.07 to 1.15, indicating the absence of collinearity problems. The ROC curves and discriminative performance of the clinicoradiological model, radiomics model, and combined model in the training set and validation set are shown in **Figures 2A, B**. The clinicoradiological model showed good predictive efficacy, and its AUC (95% CI), specificity, sensitivity and accuracy in predicting MVI were 0.778 (95% CI, 0.700 – 0.857), 63.5%, 87.1% and 77.0%, respectively, in the training set, and 0.843 (95% CI, 0.733 – 0.953), 77.8%, 84.6% and 73.6%, respectively, in the validation set. The radiomics signature showed favourable predictive performance, with an AUC of 0.896 (95% CI, 0.846 – 0.946) in the training set and 0.788 (95% CI, 0.666 – 0.910) in the validation set, and the specificity, sensitivity and accuracy were 81.0%, 82.4% and 81.8% in the training set and 55.6%, 96.2% and 66% in the validation set, respectively. The combined model showed an improved predictive performance, with AUC, specificity, sensitivity and accuracy of 0.932 (95% CI, 0.893 – 0.970), 92.1%, 80.0% and 85.1%, respectively, in the training set and 0.917 (95% CI, 0.841 – 0.994), 85.2%, 88.5% and 84.9%, respectively, in the validation set. The combined model outperformed the other two models, showing a significantly higher AUC than the clinicoradiological model and the radiomics model in the training set (*p* < 0.001, *p* = 0.041, respectively) and in the validation set (*p* = 0.035, *p* = 0.041, respectively).

**Figure 3:**
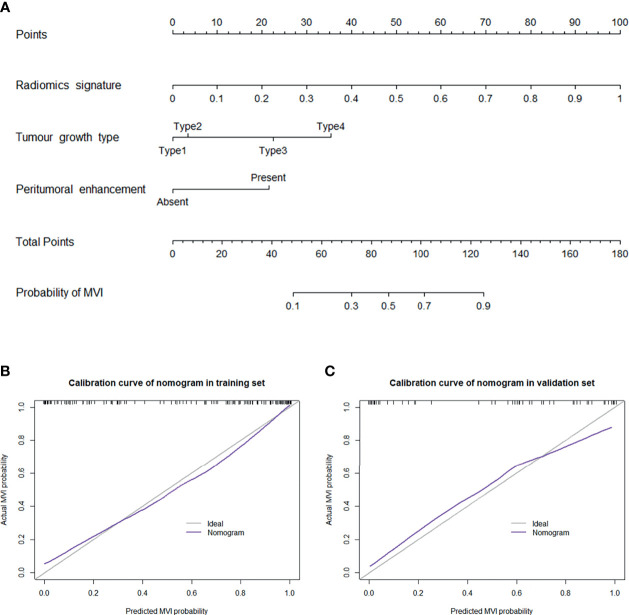
**(A)** Nomogram for the prediction of MVI presence in patients with solitary HCC. The nomogram was established based on the MR radiomics signature and 2 independent clinicopathological risk factors: peritumoral enhancement and tumour growth type (type 1: smooth regular nodule growth; type 2: focal extranodular growth; type 3: multinodular confluent growth; and type 4: infiltrative growth). Plots **(B, C)** present the calibration curve of the nomogram in the training and validation sets, respectively. The 45° gray line represents the ideal prediction, and the purple line represents the predictive performance of the nomogram. The purple line has a closer fit to the gray line, which indicates that the predicted MVI probability has good agreement with the actual presence of MVI.

**Figure 4:**
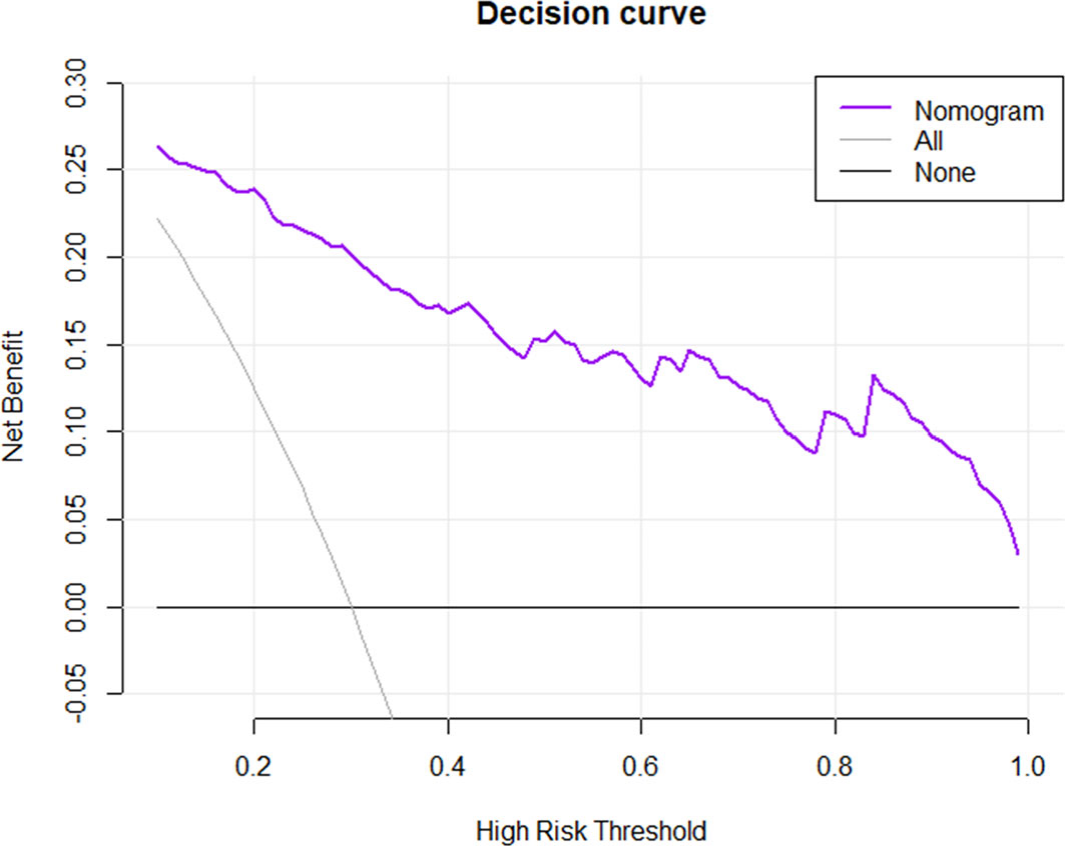
Decision curve analysis for the nomogram in predicting the presence of MVI. The net benefit was plotted versus the high-risk threshold. The purple line represents the nomogram. The gray and black lines represent the hypothesis that all patients and no patients had MVI presence, respectively.

A nomogram was established based on the combined model to individually estimate the probability of MVI in patients with solitary HCC (**Figure 3A**). The calibration curve of the nomogram showed good agreement between the predicted and actual MVI status in the training set (**Figure 3B**) and validation set (**Figure 3C**) (Hosmer–Lemeshow test, *p* = 0.412 and 0.631, respectively). The decision curve proved that using this nomogram to predict MVI adds more net benefit than either the treat-all or the treat-none scheme (**Figure 4**).

### Subgroup Analysis

The incidence of MVI was higher in the HCC > 3 cm cohort than in the HCC ≤ 3 cm cohort (68.22% *vs*. 40.43%, *p* < 0.001), which was consistent with previous studies (13). The detailed clinicoradiological characteristics of patients in the two cohorts are shown in **Supplemental Table 3**. As part of this study, the performance of clinicoradiological factors in predicting MVI was separately evaluated in the HCC ≤ 3 cm and HCC > 3 cm cohorts. Backward stepwise selection using the AIC in binary logistic regression modelling identified that tumour growth type was an independent risk factor for MVI in the HCC ≤ 3 cm cohort (**Supplemental Table 4**), while peritumoral enhancement for MVI in the HCC >3 cm cohort (**Supplemental Table 5**). The AUC (95% CI) of the clinicoradiological risk factors in predicting MVI was 0.828 (95% CI, 0.744 – 0.913) in the HCC ≤ 3 cm cohort and 0.749 (95% CI, 0.643 – 0.855) in the HCC > 3 cm cohort.

**Figure 5:**
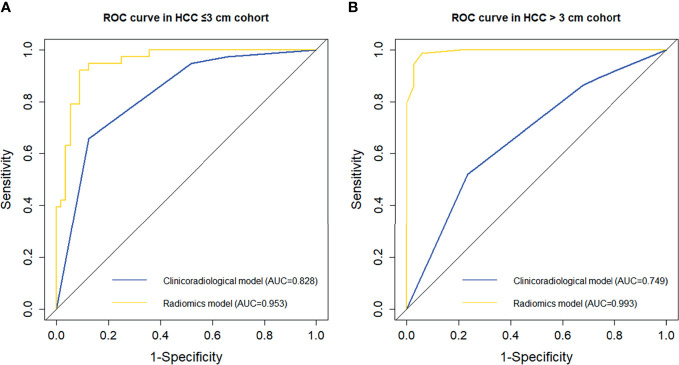
Comparison of receiver operating characteristic (ROC) curves in predicting MVI presence in the subgroups. ROC curves of the clinicoradiological model and radiomics model in the **(A)** HCC ≤ 3 cm and **(B)** HCC > 3 cm cohorts.

Subsequently, the predictive performance of radiomic features for MVI was separately evaluated in the two subgroups. The selected radiomic features for MVI prediction in the HCC ≤ 3 cm and HCC > 3 cm cohorts are separately listed in **Supplemental Tables 6** and **7**. There were more radiomic features that were associated with the presence of MVI in the HCC > 3 cm cohort than in the HCC ≤ 3 cm cohort. The performance of the ROI in each phase in the two subgroups is presented in **Supplemental Table 8** and **Figure 3**. In the HCC ≤ 3 cm cohort, the performance of the ROI was relatively stable in precontrast T_1_WI and HBP, while ROI-merge in the AP, DP, and ROI-external in the VP outperformed other ROI in their phases. Compared with the original training set, the performances of the ROI in precontrast T_1_WI and HBP were improved, while the performance of most ROI in the AP, VP and DP was decreased in the HCC ≤ 3 cm cohort. Likewise, the optimal ROI of each phase were selected to build a radiomics model. The combination of optimal ROI in each phase (i.e., ROI-merge in AP and DP, ROI-external in HBP, and ROI-plus in VP and T_1_WI) achieved an AUC of 0.953 (95% CI, 0.913 – 0.992) in the HCC ≤ 3 cm cohort, and the sensitivity, specificity and accuracy were 91.1%, 92.1% and 90.4%, respectively. In the HCC > 3 cm cohort, all the ROI showed good performance (AUC > 0.7), and all showed improvement when compared with the original training set. The combination of the optimal ROI in each phase (i.e., ROI-merge in the AP, DP and HBP, and ROI-plus in the VP and T_1_WI) obtained an AUC of 0.993 (95% CI, 0.982 – 1.000), and the sensitivity, specificity and accuracy were 94.1%, 98.6% and 97.2%, respectively, in the HCC > 3 cm cohort. The ROC curves of the clinicoradiological model and radiomics model are shown in **Figure 5**. The radiomics models were superior to the clinicoradiological models in the HCC ≤ 3 cm and > 3 cm cohorts (*p* = 0.006, *p* < 0.001, respectively).

## Discussion

The preoperative evaluation of MVI status may facilitate HCC patient management and improve survival (5). In the present study, we assessed the performance of clinicoradiological factors and radiomic features in the prediction of MVI. Our results demonstrated that clinicoradiological features, peritumoral enhancement and tumour growth type were independent risk factors for MVI; the MR radiomics signature, converted into a quantitative Rad-score, could be an independent predictor for MVI. We established and validated an MR radiomics-based nomogram for the preoperative prediction of MVI in patients with solitary HCC. Encouragingly, we further identified that the performance of clinicoradiological factors and the radiomics signature for MVI prediction varied between the HCC ≤ 3 cm and > 3 cm subgroups; the predictive performance of the radiomics signature was comparatively improved in both subgroups compared to the original training set.

Radiomics based on medical image data is a promising application in oncology. The accurate delineation of ROI is of primary importance for radiomics analysis. In the present study, the entire volumetric tumour contours were obtained using semiautomatic segmentation algorithms, with reference to the tumour boundary on HBP images. As MVI generally occurs in the peritumoral region within a 1 cm distance from tumour boundaries (8), we evaluated the performance of radiomic features extracted from both intratumoral and peritumoral areas (1 cm). Although previous studies have also established combined intratumoral and peritumoral radiomics models to predict MVI in HCC patients, the automatically extracted peritumoral areas might include extrahepatic tissues, which would result in a loss of accuracy of the models (17, 18, 21, 35). In our study, extrahepatic areas were excluded. The radiomics signature performed favourably as supported by an AUC of 0.896 in the training set. Our results showed that the different ROI (i.e., intratumoral and peritumoral areas) of phases (i.e., the precontrast T_1_WI, AP, VP, DP and HBP) can capture complementary information, thus achieving increased performance when combined.

Being partly consistent with previous studies, the features incorporated into the radiomics model also included first order features, shape-based features, and texture features (15, 18, 21, 35). For instance, the shape-based features, original shape sphericity and maximum 3D diameter, which represent spherical disproportion and the largest size of the tumour, were found to be significantly related to the presence of MVI in our study. These features were similar to the well-known independent risk features of a larger tumour diameter and nonsmooth margins (irregular growth type) for MVI (14–17). The levels of gray-level cooccurrence matric (GLCM) features are useful for quantifying the heterogeneity of tumours. In our study, the wavelet transform-based GLCM features, inverse variance, extracting from peritumoral areas, showed a strong negative correlation with MVI. Inverse variance is a parameter that reflects the degree of texture regularity; the smaller the value is, the higher the irregularity. This suggested that the presence of MVI in peritumoral areas could result in higher irregularity. Inverse variance has been proven to be useful for the discrimination between hepatic metastasis and HCC (36). In addition, the wavelet transform-based neighbouring gray-tone difference matrix (NGDTDM) feature, coarseness, was also found to be negatively correlated with MVI. Coarseness measures the average difference between the centre voxel and its neighbourhood, and indicates the level of the spatial change rate of intensity. The higher the value, the lower the spatial change rate, and the more uniform the local texture. This indicated that the presence of MVI would lead to more uneven local texture. Coarseness has been found to be clinically useful to distinguish between normal and abnormal tissue in patients with head-and-neck cancer (37). Other features have also been found to be of value and have shown promise in predicting the presence of MVI, such as the wavelet transform-based GLSZM feature (e.g., small area low gray level emphasis, SALGLE) and GLDM feature (e.g., dependence nonuniformity normalized, DNN). Based on the above explanation and analysis, our results indicated that HCC with MVI may be more likely to present as larger sizes and show irregular growth type, higher irregularity of texture, higher spatial change rate, and less uniform of local texture.

The radiomics signature based on MRI has shown promise in predicting the presence of MVI, while clinicoradiological factors may be useful for improving the predictive performance of the model. Peritumoral enhancement (13, 15–17, 38, 39) and infiltrative growth type (13, 32) have been corroborated as independent predictors for MVI, which corresponded to our results. The association of peritumoral enhancement with MVI could be explained by perfusion changes following MVI. Several studies have affirmed that peritumoral enhancement could be compensatory arterial hyperperfusion for reduced portal flow, which might be induced by minute portal branch occlusions resulting from microscopic tumour thrombi around the tumour (40, 41). Previously, “focal extranodular extension”, “focal infiltrative margin” and “multinodular confluence” tumour growth types have been reported as important predictors for MVI and showed a higher risk for MVI than a tumour with a “smooth margin and capsule” (33). In our study, infiltrative growth type was one of the independent predictors for MVI, with a strong tendency toward statistical significance (OR = 15.73). The infiltrative growth type has been reported to be associated with the metalloproteinases elaborated by HCC, which may facilitate tumour cells to infiltrate into and through the tumour capsule into the surrounding parenchyma (42), thus increasing the risk of vascular invasion. In addition, the loss of the normal expression of E-cadherin, a suppressor of cancer cell invasion, has also been reported to be associated with the infiltrative growth type (43).

Increasing research has shown that when HCC grows to a size of 3 cm, it might reach an important turning point for critical transformation with a resultant change to a more aggressive behaviour due to the changes in DNA stem lines and biological characteristics (27, 28). Sudeep et al. also pointed out that the performance of MVI predictor, radio-genomic venous invasion (RVI), varied between tumours ≤ 3 cm and >3 cm (29). Thus, in our study, the performance of clinicoradiological factors and radiomic features for MVI prediction were further separately evaluated in HCC ≤ 3 cm and >3 cm cohorts. Of note, the multivariate logistic regression analysis revealed that the tumour growth type was an independent risk factor for MVI in the HCC ≤ 3 cm cohort, while peritumoral enhancement was an independent risk factor for MVI in the HCC > 3 cm cohort. Furthermore, compared with the original training set, the performance of all ROI in HBP and precontrast T_1_WI improved in the HCC ≤ 3 cm cohort but decreased in most of the ROIs in the AP, VP and DP, while the performance of the ROI in all phases improved obviously in the HCC > 3 cm cohort. The performance of the ROI in the AP, VP and DP outperformed the ROI in HBP and precontrast T_1_WI in the HCC > 3 cm cohort. These findings suggested that the performance of the clinicoradiological factors and radiomic features for MVI prediction varied between the HCC ≤ 3 cm and HCC > 3 cm cohorts. Likewise, after the combination of the optimal ROI in each phase, the performance of the radiomics signature in MVI prediction obviously improved in both subgroups, with increases of 0.057, 10.1%, 9.7% and 8.6% in AUC, sensitivity, specificity and accuracy, respectively, in the HCC ≤ 3 cm cohort and 0.097, 13.1%,16.2% and 13.5%, respectively, in the HCC > 3 cm cohort. The improvement of prediction performance in the subgroups was most likely the result of a decrease in confounding variables between the HCC ≤ 3 cm and > 3 cm cohorts. Thus, we might safely draw the conclusion that it is necessary to group HCC by using a tumour size (3 cm) and then analyse them separately to improve the performance of MVI prediction. Unfortunately, the small number of cases prevented us from performing an internal validation analysis on the HCC ≤ 3 cm and > 3 cm cohorts. This finding is promising but premature and should be further validated in a large independent internal and external cohort of patients.

There are some underlying limitations in our study. First, the retrospective design of the present single-centre study, together with the selection bias of solitary HCC treated by surgical resection, may result in an incomplete representation of all HCC radiological features and the radiomics signature. The model was validated internally but lacked external validation. Second, the ROI were semiautomatically drawn. This could be a limiting factor because interobserver variability is known to affect results (44). However, by using the tumour boundary on HBP images as a reference for ROI segmentation, high interobserver reproducibility could be achieved. Third, the training and validation cohorts were grouped according to MRI examination time, which may cause some problems if image acquisition or surgical indications change over time. However, according to the TRIPOD statement, temporal validation is superior to random splitting (45). In our study, the radiomics model performed slightly less well in the validation set, which may be partly attributed to the differences in MR scanning instruments and parameters between the training and validation sets. Although the performance of the radiomic features extracted from the 1.5T scanner outperformed those extracted from the 3.0T scanner, there was no significant difference between them (*p* = 0.0695) in the present study, which needs further exploration. Finally, in the subgroup analysis, validation was not performed due to the small number of cases.

## Conclusion

In conclusion, our results indicated that the performance of MVI prediction in patients with solitary HCC could be improved by combining the MR radiomics signature of the optimal ROI in multiple phases. Patients with high Rad-scores may experience a higher risk of MVI. A nomogram combining the MR radiomics signature and clinicopathological risk factors may serve as an effective tool to guide the individualized management and tailored follow-up of patients with solitary HCC, although this would require further external validation prior to widespread application in clinical practice. Moreover, the performance of clinicoradiological factors and the radiomics signature for MVI prediction varied between the subgroups (HCC ≤ 3 cm and HCC > 3 cm), and the performance of the radiomics signature in MVI prediction was improved in both subgroups, which needs further internal and external validation.

## Data Availability Statement

The original contributions presented in the study are included in the article/**Supplementary Material**. Further inquiries can be directed to the corresponding authors.

## Ethics Statement

The studies involving human participants were reviewed and approved by medical ethics committee of second affiliated hospital of army medical university, PLA. Written informed consent for participation was not required for this study in accordance with the national legislation and the institutional requirements.

## Author Contributions

Study concept and design: YY, GW, and DZ. Drafting of the manuscript: YY. Review and editing of the manuscript: DZ and HL. Methodology: YY, HL, and WF. Collection and assembly of data: YY, GW, LY, TG, HC, and YL. Data analysis: YY and HL. All authors contributed to the article and approved the submitted version.

## Conflict of Interest

Author HL was employed by GE Healthcare.

The remaining authors declare that the research was conducted in the absence of any commercial or financial relationships that could be construed as a potential conflict of interest.

## Publisher’s Note

All claims expressed in this article are solely those of the authors and do not necessarily represent those of their affiliated organizations, or those of the publisher, the editors and the reviewers. Any product that may be evaluated in this article, or claim that may be made by its manufacturer, is not guaranteed or endorsed by the publisher.
