# Supplementary material for: Radiomic Features of Multi-ROI and Multi-Phase MRI for the Prediction of Microvascular Invasion in Solitary Hepatocellular Carcinoma
Source: Front Oncol. 2021 Oct 7;11:756216. doi: 10.3389/fonc.2021.756216 (PMC8529277; doi:10.3389/fonc.2021.756216)
Supplement: Supplementary file 1 [file DataSheet_1.docx]

**Supplemental Table 1.** **The selected features and their coefficients in different ROI of phases in the training set.**

| **ROI** | **Phases** | **Features** | **Coefficient values** |
| --- | --- | --- | --- |
| **Merge** | **AP** | wavelet.HLL_glszm_LargeAreaHighGrayLevelEmphasis  original_shape_Maximum3DDiameter  wavelet.LLH_glcm_MCC  original_firstorder_Skewness  wavelet.LLH_glszm_LargeAreaLowGrayLevelEmphasis  wavelet.HHH_glcm_InverseVariance  wavelet.HHH_firstorder_TotalEnergy  wavelet.HLL_firstorder_Kurtosis  wavelet.LLL_glcm_Imc2  wavelet.HHH_glrlm_LongRunLowGrayLevelEmphasis | -5.63e-8  0.03266  -20.2718  1.540586  0.000229  64.15374  -2.73e-6  -0.0882  70.23101  -0.64202 |
|  | **VP** | original_glcm_ClusterShade  wavelet.LHH_firstorder_Kurtosis  original_firstorder_10Percentile  wavelet.HHH_ngtdm_Contrast  wavelet.LHL_firstorder_Kurtosis  wavelet.HLH_firstorder_Kurtosis  wavelet.HLL_glszm_SmallAreaLowGrayLevelEmphasis | 2.92e-6  0.096908  -0.00228  -10.8732  -0.17026  -0.14632  -197.789 |
|  | **DP** | original_firstorder_10Percentile  wavelet.HHH_glszm_SizeZoneNonUniformity  wavelet.LLL_glcm_JointEnergy  wavelet.HHH_glcm_MaximumProbability  wavelet.LHL_glrlm_LongRunLowGrayLevelEmphasis  wavelet.LHL_ngtdm_Complexity  wavelet.HHH_ngtdm_Contrast  wavelet.HLH_firstorder_Kurtosis | -0.00212  -0.00148  -2195.19  49.23526  34.36017  3.78e-5  -20.0495  -0.08389 |
|  | **HBP** | wavelet.LLL_glcm_Idn | 33.7585 |
|  | **T_1_WI** | wavelet.LLL_glszm_SmallAreaLowGrayLevelEmphasis  wavelet.HHL_glcm_InverseVariance  original_ngtdm_Contrast | -252.531  -13.692  -14.953 |
| **External** | **AP** | original_shape_Maximum3DDiameter  wavelet.HHH_glcm_Correlation  wavelet.HLH_ngtdm_Contrast  wavelet.HHH_glszm_GrayLevelNonUniformity  wavelet.HHH_glszm_GrayLevelVariance  wavelet.LHH_gldm_DependenceNonUniformityNormalized | 0.02620  -27.400  86.060  -0.0044  0.4966  -116.20 |
|  | **VP** | wavelet.HHL_gldm_DependenceNonUniformityNormalized  original_shape_Maximum3DDiameter  wavelet.HHL_glszm_SmallAreaLowGrayLevelEmphasis  wavelet.LLL_glcm_ClusterProminence | -24.3678  0.011321  -24.3258  5.94e-11 |
|  | **DP** | wavelet.HLH_glcm_Correlation  wavelet.LLL_gldm_LargeDependenceLowGrayLevelEmphasis  original_shape_Maximum2DDiameterSlice  wavelet.LHL_firstorder_Kurtosis  wavelet.HHH_glszm_LargeAreaLowGrayLevelEmphasis  wavelet.LHL_glcm_Imc1  wavelet.LHL_glcm_ClusterProminence  wavelet.HLL_firstorder_Maximum | -29.9957  -1024.39  0.012813  -0.07485  -4.72e-8  -50.1684  -6.12e-7  0.003023 |
|  | **HBP** | original_ngtdm_ Joint energy  wavelet.HLH_glcm_Imc1  wavelet.LHH_glszm_LargeAreaHighGrayLevelEmphasis  wavelet.LHL_glszm_ZoneEntropy  original_shape_Sphericity | -486.795  42.15582  -4.4e-9  1.897156  -3.38493 |
|  | **T_1_WI** | wavelet.HHH _ngtdm_ Contrast  wavelet.HHL_glcm_InverseVariance  wavelet.HHH_glrlm_RunEntropy  wavelet.HLL_firstorder_Mean  wavelet.LLL_gldm_SmallDependenceLowGrayLevelEmphasis | -68.9772  -26.8915  -14.9333  0.376892  -11941.6 |
| **Plus** | **AP** | original_shape_Maximum3DDiameter  wavelet.HHH_glcm_InverseVariance  wavelet.LLH_glszm_LargeAreaLowGrayLevelEmphasis  wavelet.HHH_firstorder_TotalEnergy  wavelet.HHH_glszm_GrayLevelVariance  wavelet.HLH_glcm_Correlation  wavelet.LHH_ngtdm_Contrast  original_shape_Sphericity  wavelet.LHH_gldm_DependenceNonUniformityNormalized  wavelet.LHL_firstorder_Kurtosis  wavelet.LLH_glcm_Idmn  wavelet.LLH_glcm_Imc1  wavelet.HLL_ngtdm_Strength | 0.034002  96.62188  0.000122  -2.59e-6  0.759307  -14.3437  -40.1604  -6.02943  -135.079  -0.18649  -192.886  18.81332  -0.21763 |
|  | **VP** | wavelet.HHH_glrlm_RunPercentage  wavelet.LLH_glcm_Imc1  original_firstorder_10Percentile  original_shape_Sphericity  wavelet.HHL_gldm_SmallDependenceLowGrayLevelEmphasis  wavelet.HHH_glszm_GrayLevelVariance | -25.8265  -12.4880  -0.00129  -7.45933  -1138.28  0.33627 |
|  | **DP** | wavelet.HLH_glcm_Imc1  wavelet.LLH_glszm_LargeAreaEmphasis  wavelet.LLL_glrlm_LongRunLowGrayLevelEmphasis  wavelet.HHH_glszm_GrayLevelVariance | 44.82257  3.12e-7  -11615.9  0.204219 |
|  | **HBP** | wavelet.HLH_glszm_ZoneVariance  wavelet.HLH_glcm_Imc1  wavelet.HLL_glszm_ZoneEntropy  wavelet.LHL_glszm_LowGrayLevelZoneEmphasis  original_firstorder_10Percentile | 2.02e-8  27.63351  1.866343  -62.9388  -0.00122 |
|  | **T_1_WI** | wavelet.LHL_glcm_Idmn  wavelet.LLL_firstorder_Skewness | 134.5065  0.659473 |

Note: L represents low-pass filter, H represents low-pass filter. For example, HHL represents the intensity value resulting from directional filtering of X with a high-pass filter along x-direction, a high pass filter along y-direction and a low-pass filter along z-direction.

**Supplemental Table 2. The AUC (95% CI), sensitivity and specificity of each ROI in single phase in the training and validation sets.**

| **ROI** | **Phase** | **Training set** | | | **Validation set** | | | **Delong** |
| --- | --- | --- | --- | --- | --- | --- | --- | --- |
|  |  | **AUC (95% CI)** | **Sensitivity** | **Specificity** | **AUC (95% CI)** | **Sensitivity** | **Specificity** | ***P value*** |
| **Merge** | **AP** | 0.845(0.777-0.899) | 0.882 | 0.714 | 0.728(0.588-0.841) | 0.769 | 0.630 | 0.139 |
|  | **VP** | 0.805(0.732-0.865) | 0.682 | 0.841 | 0.731(0.591-0.843) | 0.538 | 0.889 | 0.355 |
|  | **DP** | 0.769(0.693-0.835) | 0.588 | 0.841 | 0.694(0.552-0.813) | 0.848 | 0.519 | 0.380 |
|  | **HBP** | 0.661(0.578-0.736) | 0.812 | 0.492 | 0.688(0.546-0.808) | 0.500 | 0.889 | 0.755 |
|  | **T_1_WI** | 0.702(0.621-0.774) | 0.671 | 0.667 | 0.684(0.542-0.805) | 0.759 | 0.667 | 0.837 |
| **External** | **AP** | 0.748(0.670-0.816) | 0.824 | 0.587 | 0.714(0.573-0.829) | 0.615 | 0.852 | 0.684 |
|  | **VP** | 0.740(0.662-0.809) | 0.871 | 0.556 | 0.661(0.518-0.785) | 0.462 | 0.920 | 0.372 |
|  | **DP** | 0.761(0.684-0.827) | 0.824 | 0.619 | 0.675(0.533-0.797) | 0.769 | 0.593 | 0.319 |
|  | **HBP** | 0.720(0.640-0.791) | 0.541 | 0.841 | 0.637(0.493-0.764) | 0.808 | 0.481 | 0.354 |
|  | **T_1_WI** | 0.767(0.690-0.832) | 0.835 | 0.587 | 0.638(0.495-0.766) | 0.538 | 0.926 | 0.173 |
| **Plus** | **AP** | 0.821(0.749-0.879) | 0.682 | 0.857 | 0.735(0.596-0.847) | 0.692 | 0.741 | 0.275 |
|  | **VP** | 0.741(0.662-0.809) | 0.565 | 0.794 | 0.702(0.561-0.820) | 0.709 | 0.630 | 0.648 |
|  | **DP** | 0.694(0.614-0.767) | 0.894 | 0.506 | 0.631(0.487-0.759) | 0.577 | 0.778 | 0.491 |
|  | **HBP** | 0.686(0.605-0.760) | 0.882 | 0.429 | 0.660(0.516-0.784) | 0.538 | 0.852 | 0.766 |
|  | **T_1_WI** | 0.694(0.613-0.767) | 0.565 | 0.745 | 0.651(0.508-0.777) | 0.615 | 0.778 | 0.633 |

The Delong test was applied between the training and validation sets.

**Supplemental Table 3. Patient characteristics in the HCC ≤ 3 cm and HCC＞3 cm cohorts.**

| **Variables** | **HCC ≤ 3 cm** | |  | **HCC＞3 cm** | |  | ***P value*** |
| --- | --- | --- | --- | --- | --- | --- | --- |
|  | **MVI absence**  **n=56** | **MVI presence**  **n=38** | ***P value*** | **MVI absence**  **n=34** | **MVI presence**  **n=73** | ***P value*** | <0.001 |
| Age | 51.50(47.00, 55.00) | 50.50(47.00, 57.75) | 0.568 | 49.00(43.500, 63.25) | 52.00(47.00, 62.50) | 0.217 | 0.451 |
| Sex |  |  | 0.883 |  |  | 0.876 | 0.668 |
| Female | 11(19.64%) | 7(18.42%) |  | 6(17.65%) | 12(16.44%) |  |  |
| Male | 45(80.36%) | 31(81.58%) |  | 28(82.35%) | 61(83.56%) |  |  |
| AFP level (ng/ml) |  |  | 0.397 |  |  | 0.118 | <0.001 |
| ＜20 | 28(50.00%) | 14(36.84%) |  | 17(50.00%) | 22(30.14%) |  |  |
| 20—400 | 18(32.14%) | 17(44.74%) |  | 6(17.65%) | 14(19.18%) |  |  |
| ＞400 | 10(17.86%) | 7(18.42%) |  | 11(32.35%) | 37(50.68%) |  |  |
| ALT level (IU/L) |  |  | 0.312 |  |  | 0.768 | 0.815 |
| ＜40 | 31(55.36%) | 25(65.79%) |  | 19(55.88%) | 43(58.90%) |  |  |
| ＞40 | 25(44.64%) | 13(34.21%) |  | 15(44.12%) | 30(41.10%) |  |  |
| AST level (IU/L) |  |  | 0.555 |  |  | 0.828 | 0.053 |
| ＜35 | 35(62.50%) | 26(68.42%) |  | 18(52.94%) | 37(50.68%) |  |  |
| ＞35 | 21(437.50%) | 12(31.58%) |  | 16(47.06%) | 36(49.32%) |  |  |
| ALB level (g/L) |  |  | 0.766 |  |  | 0.191 | 0.236 |
| ＞40 | 47(83.93%) | 31(81.58%) |  | 28(82.35%) | 67(91.78%) |  |  |
| ＜40 | 9(16.07%) | 7(18.42%) |  | 6(17.65%) | 6(8.22%) |  |  |
| T-BIL level (μmol/l) |  |  | 0.881 |  |  | 0.604 | 0.067 |
| ＜20 | 36(64.29%) | 25(67.78%) |  | 25(78.08%) | 57(78.08%) |  |  |
| ＞20 | 20(35.71%) | 13(34.21%) |  | 9(26.47%) | 16(21.92%) |  |  |
| ALP level (U/L) |  |  | 0.645 |  |  | 0.876 | 0.004 |
| ＜135 | 53(94.64%) | 37(97.37%) |  | 28(82.35%) | 61(83.56%) |  |  |
| ＞135 | 3(5.36%) | 1(2.63%) |  | 6(17.65%) | 12(16.44%) |  |  |
| GGT level (U/L) |  |  | 0.108 |  |  | 0.154 | <0.001 |
| ＜45 | 29(51.79%) | 26(68.42%) |  | 14(41.18%) | 20(27.40%) |  |  |
| ＞45 | 27(48.21%) | 12(31.58%) |  | 20(58.82%) | 53(72.60%) |  |  |
| PT (s) |  |  | 1.000 |  |  | 1.000 | 0.666 |
| ＜14 | 54(98.41%) | 37(97.37%) |  | 34(100.00%) | 71(97.26%) |  |  |
| ＞14 | 2(1.59%) | 1(2.63%) |  | 0(0%) | 2(2.74%) |  |  |
| **MR features** |  |  |  |  |  |  |  |
| Tumour size (cm) | 21.00(18.00,25.00) | 22.00(17.00,25.00) | 0.454 | 53.50(38.00,68.00) | 55.00(42.50,76.50) | 0.403 | <0.001 |
| Tumour growth type |  |  | <0.001 |  |  | 0.018 | 0.210 |
| Smooth regular nodule growth | 8(14.29%) | 1(2.63%) |  | 2(5.88%) | 2(2.74%) |  |  |
| Focal extranodular growth | 19(33.93%) | 1(2.63%) |  | 9(26.47%) | 8(10.96%) |  |  |
| Multinodular confluent growth | 22(39.29%) | 11(28.95%) |  | 15(44.12%) | 25(34.25%) |  |  |
| Infiltrative growth | 7(12.50%) | 25(65.79%) |  | 8(23.53%) | 38(52.05%) |  |  |
| Tumour capsule |  |  | 0.033 |  |  | 0.217 | 0.145 |
| Absent | 21(37.50%) | 15(39.47%) |  | 9(26.47%) | 30(41.10%) |  |  |
| Incomplete | 17(30.36%) | 19(50.00%) |  | 18(52.94%) | 35(47.95%) |  |  |
| Complete | 18(32.14%) | 4(10.53%) |  | 7(20.59%) | 8(10.96%) |  |  |
| Enhancement pattern |  |  | 0.123 |  |  | 1.000 | <0.001 |
| Untypical | 10(17.86%) | 12(31.58%) |  | 1(2.94%) | 3(4.11%) |  |  |
| Typical | 46(82.14%) | 26(68.42%) |  | 33(97.06%) | 70(95.89%) |  |  |
| Peritumoral enhancement |  |  | 0.013 |  |  | <0.001 | 0.001 |
| Absent | 38(67.86%) | 16(42.11%) |  | 23(67.65%) | 13(17.81%) |  |  |
| Present | 18(32.14%) | 22(57.89%) |  | 11(32.35%) | 60(82.19%) |  |  |
| HBP signal intensity |  |  | 0.194 |  |  | 0.734 | 0.276 |
| Hypointensity | 48(85.71%) | 36(94.74%) |  | 28(82.35%) | 62(84.93%) |  |  |
| Other | 8(14.29%) | 2(5.26%) |  | 6 (17.65%) | 11(15.07%) |  |  |
| Peritumoral hypointensity on HBP |  |  | 0.076 |  |  | 0.002 | <0.001 |
| Absent | 47(83.93%) | 26(68.42%) |  | 22(64.17%) | 24(32.88%) |  |  |
| Present | 9(16.07%) | 12(31.58%) |  | 12(35.29%) | 49(67.12%) |  |  |
| Intratumoral vasculature at AP |  |  | 0.092 |  |  | 0.158 | <0.001 |
| Absent | 50(89.29%) | 29(76.32%) |  | 16(47.06%) | 24(32.88%) |  |  |
| Present | 6(10.71%) | 9(23.68%) |  | 18(52.94%) | 49 (67.12%) |  |  |
| Intratumoral fat |  |  | 0.446 |  |  | 0.035 | 0.631 |
| Absent | 45(80.36%) | 28(73.68%) |  | 21(61.76%) | 59(80.82%) |  |  |
| Present | 11(19.64%) | 10(26.32%) |  | 13(38.24%) | 14(19.18%) |  |  |
| Intratumoral necrosis |  |  | 1.000 |  |  | 0.061 | <0.001 |
| Absent | 52(92.86%) | 35(92.11%) |  | 15(44.12%) | 19(26.03%) |  |  |
| Present | 4(7.14%) | 3(7.89%) |  | 19(55.88%) | 54(73.97%) |  |  |
| Intratumoral haemorrhage |  |  | 1.000 |  |  | 0.661 | <0.001 |
| Absent | 55(98.21%) | 37(97.37%) |  | 22(64.71%) | 44(60.27%) |  |  |
| Present | 1(1.79%) | 1(2.63%) |  | 12(35.29%) | 29(39.73%) |  |  |

**Abbreviations:** MVI, microvascular invasion; AFP, α-fetoprotein; ALT, alanine aminotransferase; AST, aspartate aminotransaminase; ALB, albumin; T-BIL, total bilirubin; ALP, alkaline phosphatase; GGT, γ-glutamyltransferase; PT, prothrombin time.

**Supplemental Table 4. Logistic Regression Analysis Showing the Association of Variables with MVI Presence in the HCC ≤ 3 cm cohort.**

| **Variables** | **Univariate analysis** | | **Multivariate analysis** | |
| --- | --- | --- | --- | --- |
|  | **OR (95% CI)** | ***P*-value** | **OR (95% CI)** | ***P*-value** |
| Age (year) | 1.02 (0.97–1.07) | 0.486 |  |  |
| Sex, Female vs. Male | 0.92 (0.31–2.61) | 0.883 |  |  |
| AFP level (ng/ml) |  |  |  |  |
| ＜20 | [Reference] |  |  |  |
| 20–400 | 1.89 (0.76–4.82) | 0.176 |  |  |
| ＞400 | 1.40 (0.43–4.46) | 0.570 |  |  |
| ALT level (IU/L),＜40 vs.＞40 | 0.64 (0.27–1.50) | 0.313 |  |  |
| AST level (IU/L),＜35 vs.＞35 | 0.77 (0.32–1.82) | 0.555 |  |  |
| ALB level (g/L),＞40 vs.＜40 | 1.18 (0.39–3.49) | 0.766 |  |  |
| T-BIL level (μmol/l),＜20 vs.＞20 | 0.94 (0.39–2.21) | 0.881 |  |  |
| ALP level (U/L),＜135 vs.＞135 | 0.48 (0.02–3.89) | 0.529 |  |  |
| GGT level (U/L),＜45 vs.＞45 | 0.50 (0.20–1.16) | 0.110 |  |  |
| PT (s),＜14 vs.＞14 | 0.73 (0.03–7.89) | 0.800 |  |  |
| **MR features** |  |  |  |  |
| Tumour size (cm) | 1.04 (0.96–1.13) | 0.325 |  |  |
| Tumour growth type |  |  |  |  |
| Smooth regular nodule growth | [Reference] |  | [Reference] |  |
| Focal extranodular growth | 0.42 (0.02–11.53) | 0.558 | 0.42 (0.02–11.53) | 0.558 |
| Multinodular confluent growth | 4.00 (0.62–79.08) | 0.217 | 4.00(0.62–79.08) | 0.217 |
| Infiltrative growth | 28.57 (4.26–579.43) | 0.003 | 28.57(4.26–579.43) | 0.003 |
| Capsule |  |  |  |  |
| Complete | [Reference] |  |  |  |
| Incomplete | 5.03 (1.52–20.13) | 0.012 |  |  |
| Absent | 3.21 (0.96–12.87) | 0.072 |  |  |
| Enhancement pattern,  Untypical vs. Typical | 0.47 (0.18–1.24) | 0.127 |  |  |
| Peritumoral enhancement,  Absent vs. Present | 2.90 (1.25–6.94) | 0.014 |  |  |
| HBP signal intensity,  Hypointensity vs. Other | 3.00 (0.70–20.68) | 0.181 |  |  |
| Peritumoral hypointensity on HBP,  Absent vs. Present | 2.41 (0.90–6.65) | 0.081 |  |  |
| Intratumoral vasculature,  Absent vs. Present | 2.59 (0.85–8.42) | 0.099 |  |  |
| Intratumoral fat,  Absent vs. Present | 1.46 (0.54–3.91) | 0.447 |  |  |
| Intratumoral necrosis,  Absent vs. Present | 1.11 (0.21–5.35) | 0.892 |  |  |
| Intratumoral haemorrhage,  Absent vs. Present | 1.49 (0.06–38.38) | 0.782 |  |  |

**Abbreviations:** MVI, microvascular invasion; OR, odds ratio; AFP, α-fetoprotein; ALT, alanine aminotransferase; AST, aspartate aminotransaminase; ALB, albumin; T-BIL, total bilirubin; ALP, alkaline phosphatase; GGT, γ-glutamyltransferase; PT, prothrombin time.

**Supplemental Table 5. Logistic Regression Analysis Showing the Association of Variables with MVI Presence in the HCC＞3 cm cohort.**

| **Variables** | **Univariate analysis** | | **Multivariate analysis** | |
| --- | --- | --- | --- | --- |
|  | **OR (95% CI)** | ***P*-value** | **OR (95% CI)** | ***P-*value** |
| Age (year) | 1.03 (0.99–1.07) | 0.120 |  |  |
| Sex, Female vs. Male | 0.92 (0.32–2.86) | 0.876 |  |  |
| AFP level (ng/ml) |  |  |  |  |
| ＜20 | [Reference] |  |  |  |
| 20–400 | 1.80 (0.59–6.00) | 0.314 |  |  |
| ＞400 | 2.60 (1.04—6.70) | 0.043 |  |  |
| ALT level (IU/L),＜40 vs.＞40 | 0.88 (0.39–2.03) | 0.768 |  |  |
| AST level (IU/L),＜35 vs.＞35 | 1.09 (0.48–2.49) | 0.828 |  |  |
| ALB level (g/L),＞40 vs.＜40 | 0.42 (0.12–1.44) | 0.159 |  |  |
| T-BIL level (μmol/l),＜20 vs.＞20 | 0.78 (0.31–2.06) | 0.605 |  |  |
| ALP level (U/L),＜135 vs.＞135 | 0.92 (0.32–2.86) | 0.876 |  |  |
| GGT level (U/L),＜45 vs.＞45 | 1.86 (0.78–4.37) | 0.157 |  |  |
| PT (s),＜14 vs.＞14 | NA | 0.989 |  |  |
| **MR features** |  |  |  |  |
| Tumour size (cm) | 1.01 (0.99–1.03) | 0.279 |  |  |
| Tumour growth type |  |  |  |  |
| Smooth regular nodule growth | [Reference] |  |  |  |
| Focal extranodular growth | 0.89 (0.09–8.88) | 0.916 |  |  |
| Multinodular confluent growth | 1.67 (0.18–15.11) | 0.627 |  |  |
| Infiltrative growth | 4.75 (0.51–44.79) | 0.146 |  |  |
| Capsule |  |  |  |  |
| Complete | [Reference] |  |  |  |
| Incomplete | 1.70 (0.52–5.50) | 0.370 |  |  |
| Absent | 2.92 (0.82–10.52) | 0.096 |  |  |
| Enhancement pattern,  Untypical vs. Typical | 0.71 (0.03–5.76) | 0.768 |  |  |
| Peritumoral enhancement,  Absent vs. Present | 9.65 (3.90–25.58) | <0.001 | 9.65 (3.90–25.58) | <0.001 |
| HBP signal intensity,  Hypointensity vs. Other | 1.21 (0.38–3.51) | 0.734 |  |  |
| Peritumoral hypointensity on HBP,  Absent vs. Present | 3.74 (1.62–9.04) | 0.003 |  |  |
| Intratumoral vasculature,  Absent vs. Present | 1.81 (0.79–4.19) | 0.160 |  |  |
| Intratumoral fat,  Absent vs. Present | 0.38 (0.15–0.95) | 0.038 |  |  |
| Intratumoral necrosis,  Absent vs. Present | 2.24 (0.95–5.31) | 0.064 |  |  |
| Intratumoral haemorrhage,  Absent vs. Present | 1.21 (0.52–2.87) | 0.661 |  |  |

**Abbreviations:** MVI, microvascular invasion; OR, odds ratio; AFP, α-fetoprotein; ALT, alanine aminotransferase; AST, aspartate aminotransaminase; ALB, albumin; T-BIL, Total bilirubin; ALP, alkaline phosphatase; GGT, γ-glutamyltransferase; PT, prothrombin time.

**Supplemental Table 6. The selected features and their coefficients in the different ROI of phases in the HCC ≤ 3 cm cohort.**

| **ROI** | **Phases** | **Features** | **Coefficient values** |
| --- | --- | --- | --- |
| **Merge** | **AP** | wavelet.HHH_glcm_InverseVariance  original_firstorder_Skewness  wavelet.HLL_glszm_LargeAreaHighGrayLevelEmphasis  wavelet.HLH_gldm_DependenceVariance  original_shape_Maximum3DDiameter  wavelet.LHL_firstorder_Median  wavelet.LLL_gldm_LargeDependenceLowGrayLevelEmphasis  wavelet.LLH_glcm_MCC  original_glcm_ClusterShade  wavelet.HLH_glcm_Imc1  wavelet.LHL_glszm_LowGrayLevelZoneEmphasis  wavelet.HHL_firstorder_Kurtosis  wavelet.HHH_glrlm_LowGrayLevelRunEmphasis | 84.4121  2.45257  2.539e-05  0.138275  0.055368  0.117041  -550.074  -25.874  -3.19e-06  -41.902  206.181  -1.21939  -12.9202 |
|  | **VP** | wavelet.LHH_firstorder_Kurtosis  wavelet.HHH_ngtdm_Contrast | 0.12015  -9.8141 |
|  | **DP** | wavelet.LHH_gldm_LargeDependenceLowGrayLevelEmphasis  wavelet.LLL_firstorder_Minimum  wavelet.LHH_firstorder_Kurtosis  wavelet.HHH_firstorder_Energy  wavelet.HHL_glcm_InverseVariance  original_glszm_LargeAreaHighGrayLevelEmphasis  original_glcm_MCC | -0.04077  -0.00129  0.16221  -0.00023  -12.6473  1.68e-05  -16.256 |
|  | **HBP** | wavelet.LLH_glcm_Imc1  original_glcm_MCC  wavelet.LLL_firstorder_Skewness  wavelet.HHL_glcm_Correlation  wavelet.HHL_glszm_SmallAreaEmphasis | -10.7158  -8.9509  1.5967  -7.145  -7.7988 |
|  | **T_1_WI** | wavelet.HHL_ngtdm_Contrast  wavelet.LHL_glcm_MCC  wavelet.HHH_gldm_LowGrayLevelEmphasis  wavelet.HHL_gldm_SmallDependenceLowGrayLevelEmphasis  wavelet.HHL_glszm_SmallAreaEmphasis | -46.6493  -8.14266  7.69757  -195.68  10.0232 |
| **External** | **AP** | original_shape_Maximum3DDiameter  wavelet.HLL_gldm_DependenceNonUniformity  wavelet.HHH_glszm_LargeAreaEmphasis  wavelet.HHL_glcm_MCC | 0.122613  -0.00033  -5.81e-08  -7.403743 |
|  | **VP** | original_shape_Maximum3DDiameter  wavelet.HHL_gldm_SmallDependenceLowGrayLevelEmphasis | 0.04927  -1033.02 |
|  | **DP** | wavelet.HHL_glcm_Correlation  wavelet.LHH_gldm_SmallDependenceLowGrayLevelEmphasis  wavelet.LHL_firstorder_Kurtosis | -10.8722  840.6782  0.1759 |
|  | **HBP** | wavelet.HHL_glcm_Correlation  wavelet.LHH_glrlm_RunEntropy  wavelet.HLL_gldm_LargeDependenceHighGrayLevelEmphasis  wavelet.HLL_glrlm_LongRunLowGrayLevelEmphasis  original_glcm_ClusterShade | -13.494  -5.2605  -3.01e-05  -141.6  -1.10e-06 |
|  | **T_1_WI** | wavelet.LHH_glcm_Imc2  wavelet.LHL_firstorder_Kurtosis  wavelet.LLH_glcm_Imc1  original_shape_Sphericity  wavelet.HHL_firstorder_Kurtosis  wavelet.HHL_glszm_SmallAreaEmphasis  wavelet.HLL_glcm_Correlation  wavelet.HHH_glszm_LargeAreaHighGrayLevelEmphasis  wavelet.HHH_glrlm_ShortRunEmphasis | -31.335  0.51995  -21.8999  -13.4016  -0.3073  9.84411  11.1981  -6.40e-09  -102.8623 |
| **Plus** | **AP** | wavelet.HHL_glcm_Correlation | -10.4066 |
|  | **VP** | original_shape_Sphericity  original_shape_Maximum3DDiameter  wavelet.HHL_glrlm_ShortRunLowGrayLevelEmphasis  wavelet.LLH_glcm_Imc1  wavelet.HHL_glcm_Correlation  original_glcm_Idmn  wavelet.HHL_firstorder_Kurtosis | -6.1495  0.0769  -27.464  -21.2629  -6.7015  -405.4  0.0754 |
|  | **DP** | wavelet.HHL_glcm_Correlation | -11.5706 |
|  | **HBP** | wavelet.HHL_glcm_Correlation  wavelet.LHH_glrlm_RunEntropy  original_ngtdm_Busyness | -11.5092  -5.0684  -2.6609 |
|  | **T_1_WI** | original_shape_Sphericity  wavelet.HLL_glszm_LargeAreaLowGrayLevelEmphasis  wavelet.LHH_glcm_Imc2  wavelet.LHL_glcm_Idmn  original_gldm_SmallDependenceLowGrayLevelEmphasis  wavelet.HHL_glszm_SizeZoneNonUniformityNormalized  wavelet.LHL_firstorder_Median  wavelet.HHL_glcm_MCC | -19.5293  -0.0001  -18.7455  423.4356  -5736.87  10.09295  -0.81887  -17.4970 |

**Note:** L represents low-pass filter, H represents low-pass filter. For example, HHL represents the intensity value resulting from directional filtering of X with a high-pass filter along x-direction, a high pass filter along y-direction and a low-pass filter along z-direction.

**Supplemental Table 7.The selected features and their coefficients in the different ROI of phases in the HCC＞3 cm cohort**

| **ROI** | **Phases** | **Features** | **Coefficient values** |
| --- | --- | --- | --- |
| **Merge** | **AP** | wavelet.LLL_glcm_ClusterShade  original_shape_Sphericity  wavelet.HHH_glcm_Correlation  original_firstorder_10Percentile  wavelet.LHL_firstorder_Median  original_glszm_LargeAreaHighGrayLevelEmphasis  wavelet.LHL_glrlm_LongRunHighGrayLevelEmphasis  wavelet.LLL_glcm_Correlation  wavelet.HLL_firstorder_Kurtosis  wavelet.LLH_glcm_Imc1  wavelet.HLL_glcm_Imc1  original_gldm_LargeDependenceLowGrayLevelEmphasis | 1.33e-07  -5.87615  -78.893  -0.0042  0.47155  -2.94e-06  -0.00017  23.525  -0.0851  99.2814  -60.3676  -118.8737 |
|  | **VP** | wavelet.LLL_glcm_ClusterShade  original_shape_Sphericity  original_firstorder_10Percentile  wavelet.HHL_glcm_Correlation  wavelet.LLH_glcm_Imc1  wavelet.LHH_firstorder_Kurtosis  wavelet.HLL_glcm_Imc1  wavelet.HHL_glrlm_LongRunHighGrayLevelEmphasis  wavelet.HLL_glszm_LargeAreaHighGrayLevelEmphasis  wavelet.HLL_glcm_Idmn  wavelet.LLL_glcm_Imc2  wavelet.LLL_glszm_LargeAreaLowGrayLevelEmphasis | 2.73e-07  -4.8676  -0.0055  25.0002  30.5001  0.14212  59.9718  -0.0014  -5.68e-08  -289.104  59.4422  -308.243 |
|  | **DP** | original_shape_Sphericity  wavelet.HLL_glszm_LargeAreaLowGrayLevelEmphasis  original_glcm_Imc2  wavelet.HHH_glszm_SizeZoneNonUniformity  wavelet.LHH_glcm_Correlation  wavelet.LHL_ngtdm_Busyness  wavelet.HHL_glcm_MCC  wavelet.HLL_gldm_SmallDependenceHighGrayLevelEmphasis  wavelet.LLH_glcm_MCC  wavelet.HLL_firstorder_Kurtosis  wavelet.HHL_glcm_Correlation  wavelet.LLL_glcm_Imc1  original_firstorder_Minimum  wavelet.HLH_glcm_Imc1  wavelet.HHL_glszm_LargeAreaHighGrayLevelEmphasis | -4.6841  -0.4492  -89.2195  -0.0037  -28.132  -0.2289  -8.7161  0.0017  -13.98  -0.2573  28.812  -25.577  -0.0073  123.95  1.95e-09 |
|  | **HBP** | original_shape_Sphericity  wavelet.LHL_gldm_LargeDependenceHighGrayLevelEmphasis  wavelet.HLH_glszm_LargeAreaLowGrayLevelEmphasis  wavelet.HLL_glszm_LargeAreaLowGrayLevelEmphasis  wavelet.HLH_glcm_Imc1  wavelet.LLL_glcm_ClusterProminence  wavelet.LLH_glcm_MCC  wavelet.HHL_glcm_MCC  wavelet.HHH_glszm_SizeZoneNonUniformity  wavelet.HHH_glcm_InverseVariance  wavelet.LHL_glcm_MCC  original_glszm_LargeAreaEmphasis | -8.0184  4.05e-05  1.28e-07  0.1274  103.42  -3.34e-11  -15.78  -16.003  0.0018  -70.944  20.934  -0.008 |
|  | **T_1_WI** | original_shape_Sphericity  wavelet.HHH_glcm_MaximumProbability  wavelet.HLH_glcm_Correlation  wavelet.LHL_gldm_LargeDependenceHighGrayLevelEmphasis | -5.703  87.863  -12.8261  3.92e-05 |
| **External** | **AP** | wavelet.HHH_glrlm_RunVariance  wavelet.HLL_firstorder_Mean  wavelet.LHH_firstorder_Kurtosis  wavelet.HLH_glcm_Imc1  wavelet.LLH_glcm_MCC  wavelet.HLL_glszm_LargeAreaLowGrayLevelEmphasis  wavelet.LHL_glcm_Correlation  wavelet.HHL_glcm_MCC  wavelet.LLH_glcm_Imc1 | -8.662  -0.265  0.0845  169.39  18.385  -1.813  14.368  -5.265  47.841 |
|  | **VP** | wavelet.HLL_glcm_Idmn  wavelet.HHH_gldm_DependenceNonUniformityNormalized  wavelet.HHL_gldm_DependenceNonUniformityNormalized  wavelet.LHH_firstorder_Kurtosis | -81.952  -124.379  -47.355  0.1087 |
|  | **DP** | original_glszm_ZoneEntropy  wavelet.HLL_ngtdm_Strength  wavelet.HHH_glszm_LargeAreaLowGrayLevelEmphasis  wavelet.HLH_firstorder_Kurtosis  wavelet.HHL_gldm_DependenceVariance  wavelet.LLL_firstorder_Minimum  wavelet.HLL_firstorder_Median  wavelet.HLL_glcm_Imc1  wavelet.HLL_firstorder_Kurtosis  wavelet.LLL_firstorder_InterquartileRange  wavelet.HLH_glrlm_LongRunHighGrayLevelEmphasis  wavelet.LHL_glszm_LargeAreaHighGrayLevelEmphasis | -2.4847  1.852  -4.49e-08  0.1535  0.5400  -0.001  0.6186  174.96  -0.197  0.0063  -0.0063  -1.16e-07 |
|  | **HBP** | wavelet.LHL_glcm_Idn  wavelet.HLL_firstorder_Kurtosis  wavelet.LLH_glcm_MCC  original_gldm_SmallDependenceLowGrayLevelEmphasis  wavelet.LLL_glcm_ClusterShade  wavelet.LHL_glrlm_ShortRunLowGrayLevelEmphasis  wavelet.HLL_glrlm_LongRunHighGrayLevelEmphasis  wavelet.LHH_glszm_ZoneEntropy | 94.698  -0.4728  -11.424  18186.9  4.49e-08  1671.21  0.0002  1.0927 |
|  | **T_1_WI** | wavelet.HHH_glrlm_RunVariance | -10.912 |
| **Plus** | **AP** | original_shape_Sphericity  wavelet.HLH_glcm_Imc1  original_glcm_Idmn  wavelet.HLL_firstorder_Median  wavelet.HLL_glrlm_LongRunHighGrayLevelEmphasis  wavelet.LHL_glcm_Correlation  wavelet.HHH_ngtdm_Contrast  original_shape_Maximum3DDiameter  original_glszm_LargeAreaHighGrayLevelEmphasis | -7.093  92.394  -1217.98  0.5681  -8.51e-05  9.4285  -41.72  0.0199  -2.15e-06 |
|  | **VP** | original_shape_Sphericity  wavelet.HHL_glcm_Correlation  original_ngtdm_Contrast  original_glszm_LargeAreaHighGrayLevelEmphasis  wavelet.HLH_firstorder_Kurtosis  wavelet.HHL_firstorder_Kurtosis  wavelet.LLL_firstorder_Minimum  wavelet.HLL_gldm_LargeDependenceHighGrayLevelEmphasis  wavelet.LLH_glcm_Imc1  wavelet.LLL_glszm_LargeAreaLowGrayLevelEmphasis  wavelet.LLL_glcm_Imc2  wavelet.HHL_glszm_LargeAreaHighGrayLevelEmphasis  wavelet.LHL_glcm_Correlation  wavelet.LLL_glcm_ClusterProminence  original_firstorder_10Percentile  wavelet.HLL_glcm_Idmn | -8.9402  46.936  -21.391  4.30e-07  0.0473  -0.268  0.00399  1.52e-05  56.1905  -11053.49  250.768  1.05e-09  -18.407  2.30e-11  -0.01004  -1406.76 |
|  | **DP** | original_shape_Sphericity  wavelet.LHH_glcm_Correlation  wavelet.LHH_glszm_ZonePercentage  wavelet.HLL_firstorder_Mean  original_glszm_LargeAreaHighGrayLevelEmphasis  original_firstorder_Minimum  wavelet.LLH_glcm_Imc1  wavelet.HLL_glcm_ClusterProminence  wavelet.LLL_ngtdm_Strength  wavelet.HLL_firstorder_Median  original_glcm_Idmn | -6.3923  -19.403  -140.723  0.888  -1.07e-06  -0.0065  21.916  8.62e-07  0.0074  -1.702  -1876.3 |
|  | **HBP** | wavelet.LHL_glszm_ZoneEntropy  wavelet.HHH_glszm_LargeAreaLowGrayLevelEmphasis  original_shape_Sphericity  wavelet.LLH_glcm_Imc1  wavelet.LLL_glcm_ClusterProminence | 4.596  1.38e-08  -6.881  20.761  -5.69e-12 |
|  | **T_1_WI** | wavelet.LHL_glcm_MCC  wavelet.HLL_firstorder_Mean  original_shape_Sphericity  wavelet.LHL_glcm_Correlation  original_gldm_SmallDependenceLowGrayLevelEmphasis  wavelet.LHL_firstorder_Median  wavelet.HLH_firstorder_Kurtosis  wavelet.LHH_firstorder_Kurtosis  wavelet.LHH_glszm_LargeAreaHighGrayLevelEmphasis  wavelet.HLH_glcm_Correlation  wavelet.HLL_firstorder_Median  wavelet.LHL_firstorder_Mean | 9.335  1.392  -9.167  10.325  20925.03  4.0732  0.0801  -0.0317  1.12e-10  -42.093  -2.9134  -3.1313 |

**Note:** L represents low-pass filter, H represents low-pass filter. For example, HHL represents the intensity value resulting from directional filtering of X with a high-pass filter along x-direction, a high pass filter along y-direction and a low-pass filter along z-direction.

**Supplemental Table 8. The AUC (95% CI), sensitivity and specificity of the different ROI models in the HCC ≤ 3 cm and****＞3 cm cohorts.**

| **ROI** | **Phases** | **HCC ≤ 3 cm** | | | **HCC＞3 cm** | | |
| --- | --- | --- | --- | --- | --- | --- | --- |
|  |  | **AUC (95% CI)** | **Sensitivity** | **Specificity** | **AUC (95% CI)** | **Sensitivity** | **Specificity** |
| **Merge** | **AP** | 0.935 (0.890, 0.981) | 0.895 | 0.839 | 0.889 (0.827, 0.951) | 0.890 | 0.735 |
|  | **VP** | 0.692 (0.585, 0.799) | 0.661 | 0.684 | 0.892 (0.828, 0.956) | 0.877 | 0.794 |
|  | **DP** | 0.820 (0.736, 0.905) | 0.763 | 0.786 | 0.883 (0.813, 0.953) | 0.890 | 0.735 |
|  | **HBP** | 0.739 (0.637, 0.842) | 0.553 | 0.857 | 0.855 (0.781, 0.928) | 0.781 | 0.824 |
|  | **T_1_WI** | 0.816 (0.728, 0.905) | 0.789 | 0.696 | 0.757 (0.653, 0.861) | 0.795 | 0.647 |
| **External** | **AP** | 0.742 (0.641, 0.843) | 0.447 | 0.929 | 0.813 (0.723, 0.904) | 0.959 | 0.529 |
|  | **VP** | 0.651 (0.541, 0.761) | 0.815 | 0.518 | 0.748 (0.652, 0.844) | 0.493 | 0.912 |
|  | **DP** | 0.741 (0.639, 0.843) | 0.737 | 0.750 | 0.862 (0.786, 0.938) | 0.822 | 0.794 |
|  | **HBP** | 0.780 (0.687, 0.873) | 0.605 | 0.857 | 0.795 (0.706, 0.885) | 0.753 | 0.765 |
|  | **T_1_WI** | 0.852 (0.775, 0.928) | 0.763 | 0.857 | 0.707 (0.606, 0.809) | 0.658 | 0.824 |
| **Plus** | **AP** | 0.657 (0.543, 0.771) | 0.763 | 0.607 | 0.831 (0.748, 0.915) | 0.630 | 0.912 |
|  | **VP** | 0.784 (0.692, 0.876) | 0.895 | 0.589 | 0.901 (0.842, 0.959) | 0.836 | 0.824 |
|  | **DP** | 0.656 (0.544, 0.767) | 0.711 | 0.625 | 0.807 (0.713, 0.902) | 0.740 | 0.794 |
|  | **HBP** | 0.727 (0.626, 0.828) | 0.895 | 0.482 | 0.720 (0.633, 0.817) | 0.507 | 0.882 |

|  | **T_1_WI** | 0.874 (0.802, 0.946) | 0.816 | 0.821 | 0.850 (0.769, 0.931) | 0.781 | 0.824 |
| --- | --- | --- | --- | --- | --- | --- | --- |

**Supplemental Formula 1:**

Calculation formula for AP merge rad-score:

AP merge rad-score = 2.604e+00

+ (2.330e-08) * wavelet.HLL_glszm_LargeAreaHighGrayLevelEmphasis

+ (-5.656e-03) * original_shape_Maximum3DDiameter

+ (-9.537e-01) * wavelet.LLH_glcm_MCC

+ (4.926e-01) * original_firstorder_Skewness

+ (8.946e-06) * wavelet.LLH_glszm_LargeAreaLowGrayLevelEmphasis

+ (-8.099e+00) * wavelet.HHH_glcm_InverseVariance

+ (1.421e-06) * wavelet.HHH_firstorder_TotalEnergy

+ (7.702e-02) * wavelet.HLL_firstorder_Kurtosis

+ (2.259e+00) * wavelet.LLL_glcm_Imc2

+ (-2.358e-01) * wavelet.HHH_glrlm_LongRunLowGrayLevelEmphasis

Calculation formula for VP merge rad-score:

VP merge rad-score = 4.034

+ (2.917e-06) * original_glcm_ClusterShade

+ (9.691e-02) * wavelet.LHH_firstorder_Kurtosis

+ (-2.284e-03) * original_firstorder_10Percentile

+ (-1.087e+01) * wavelet.HHH_ngtdm_Contrast

+ (-1.703e-01) * wavelet.LHL_firstorder_Kurtosis

+ (-1.463e-01) * wavelet.HLH_firstorder_Kurtosis

+ (-1.978e+02) * wavelet.HLL_glszm_SmallAreaLowGrayLevelEmphasis

Calculation formula for DP merge rad-score:

DP merge rad-score = -10.08

+ (-2.123e-03) * original_firstorder_10Percentile

+ (-1.481e-03) * wavelet.HHH_glszm_SizeZoneNonUniformity

+ (-2.195e+03) * wavelet.LLL_glcm_JointEnergy

+ (4.924e+01) * wavelet.HHH_glcm_MaximumProbability

+ (3.436e+01) * wavelet.LHL_glrlm_LongRunLowGrayLevelEmphasis

+ (3.776e-05) * wavelet.LHL_ngtdm_Complexity

+ (-2.005e+01) * wavelet.HHH_ngtdm_Contrast

+ (-8.389e-02) * wavelet.HLH_firstorder_Kurtosis

Calculation formula for HBP externa rad-score:

HBP external rad-score = -9.039

+ (-4.868e+02) * original_ngtdm_Coarseness

+ (4.216e+01) * wavelet.HLH_glcm_Imc1

+ (-4.400e-09) * wavelet.LHH_glszm_LargeAreaHighGrayLevelEmphasis

+ (1.897e+00) * wavelet.LHL_glszm_ZoneEntropy

+ (-3.385e+00) * original_shape_Sphericity

Calculation formula for T_1_WI external rad-score:

T1WI external rad-score = 56.91

+ (-6.898e+01) * wavelet.HHL_ngtdm_Contrast

+ (-2.689e+01) * wavelet.HHL_glcm_InverseVariance

+ (-1.493e+01) * wavelet.HHH_glrlm_RunEntropy

+ (3.769e-01) * wavelet.HLL_firstorder_Mean

+ (-1.194e+04) * wavelet.LLL_gldm_SmallDependenceLowGrayLevelEmphasis

Calculation formula for Radiomics score:

Radiomics score = -0.34846

+0.72433 * AP merge rad-score

+0.52206 * VP merge rad-score

+0.30080 * DP merge rad-score

+0.03609 * HBP external rad-score

+0.49454 * T_1_WI external rad-score

**Supplemental Figure Legend:**

**Supplemental Figure 1.** The least absolute shrinkage and selection operator (LASSO) logistic regression algorithm was employed to determine the significant radiomics features in ROI of each phase related to microvascular invasion (MVI) presence. (a, b, c) Selection of the tuning parameter (λ) using 10-fold cross validation. (d, e, f) LASSO coefficient profiles of the radiomics features. (a, d) ROI-merge; (b, c) ROI-external; (c, f) ROI-plus. (AP, arterial phase; VP, portal venous phase; DP, delayed phase; HBP, hepatobiliary phase; and T_1_WI, pre-contrast T_1_WI).

**Supplemental Figure 2.** ROC curves of the different ROI models in the training and validation sets.

**Supplemental Figure 3.** ROC curves of the different ROI models in the HCC ≤ 3 cm and HCC＞3 cm cohorts.
