# Supplementary figures and images for: Radiomic Features of Multi-ROI and Multi-Phase MRI for the Prediction of Microvascular Invasion in Solitary Hepatocellular Carcinoma
Source: Front Oncol. 2021 Oct 7;11:756216. doi: 10.3389/fonc.2021.756216 (PMC8529277; doi:10.3389/fonc.2021.756216)

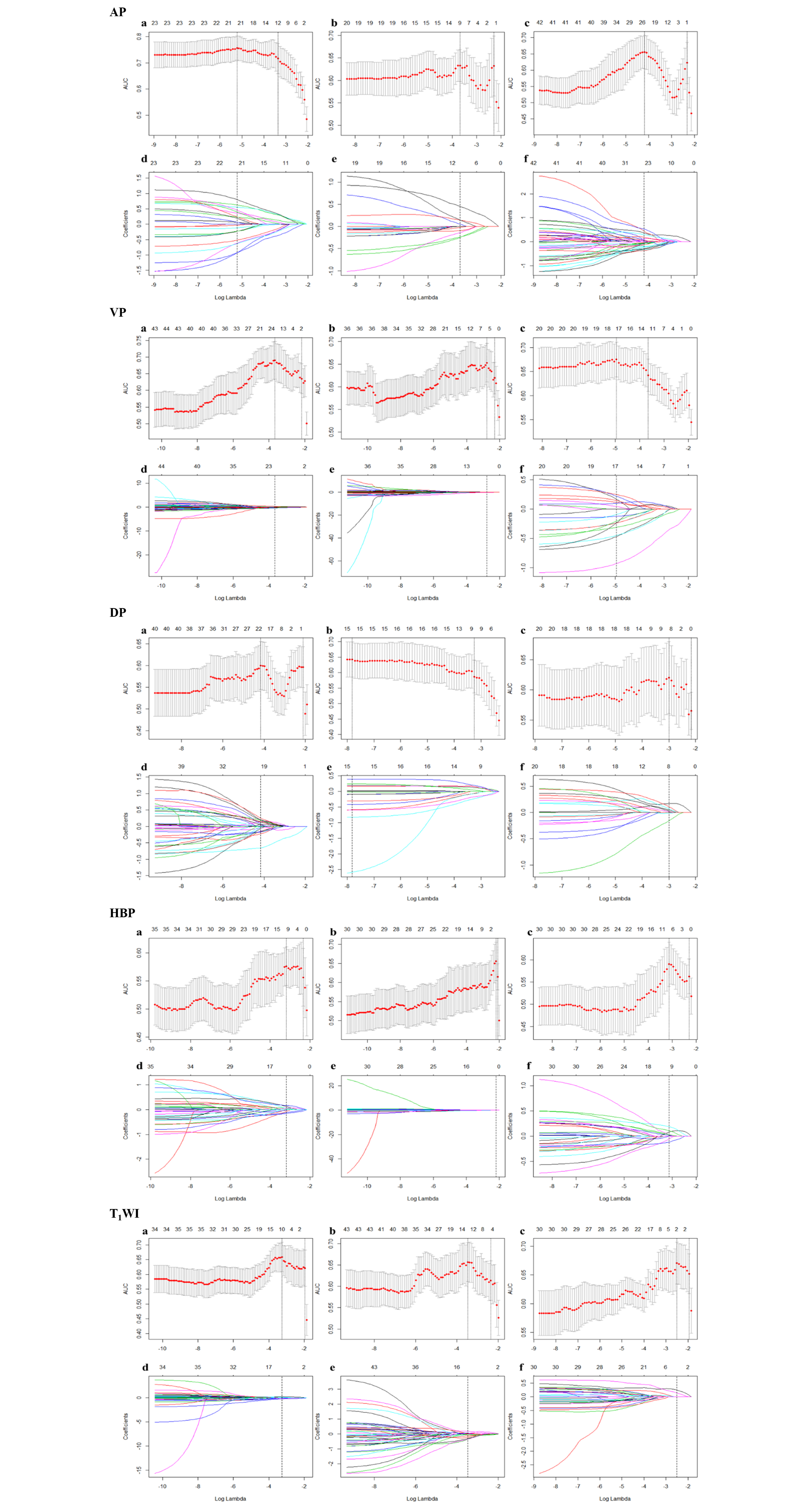

Supplement: Supplementary file 2 [file Image_1.tif]

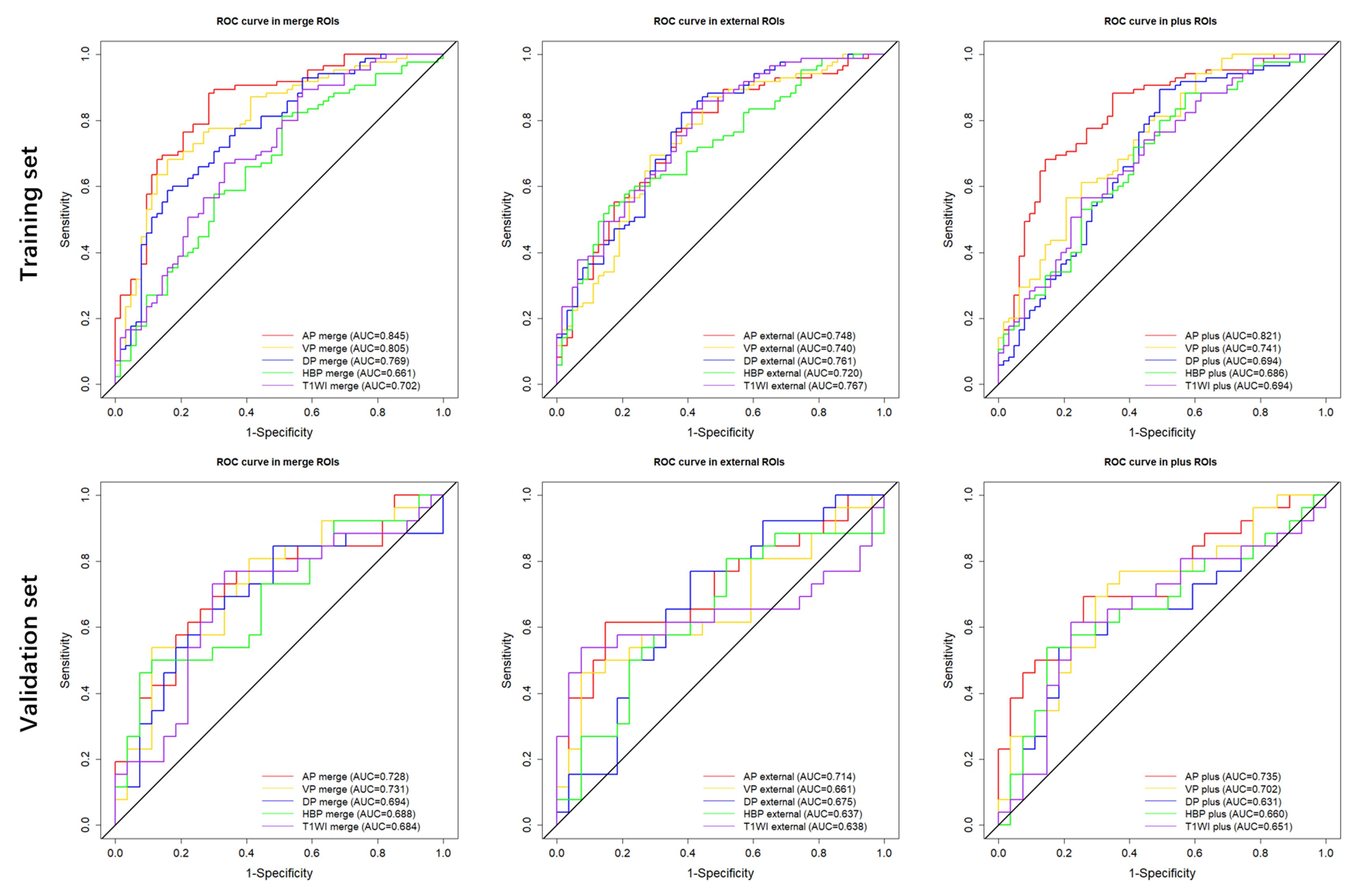

Supplement: Supplementary file 3 [file Image_2.tif]

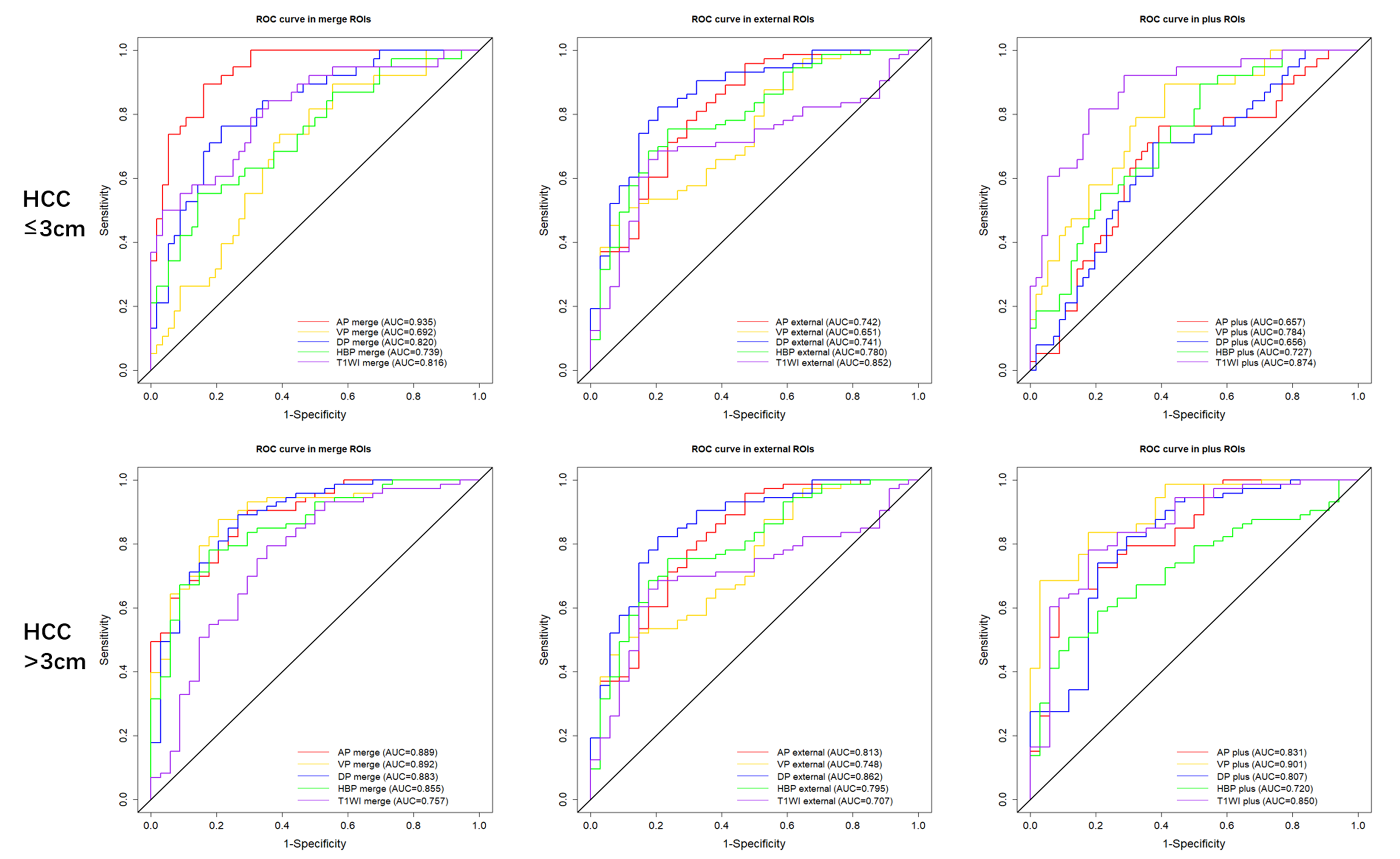

Supplement: Supplementary file 4 [file Image_3.tif]
